# Supplementary material for: Longitudinal symptom profile of palliative care patients receiving a nurse-led end-of-life (PEACH) programme to support preference to die at home
Source: BMJ Open. 2024 Jan 2;14(1):e058448. doi: 10.1136/bmjopen-2021-058448 (PMC10773358; doi:10.1136/bmjopen-2021-058448)
Supplement: Author's manuscript [file bmjopen-2021-058448.draft_revisions.pdf]

BMJ Open

BMJ Open is committed to open peer review. As part of this commitment we make the peer review history of every article we publish publicly available.

When an article is published we post the peer reviewers' comments and the authors' responses online. We also post the versions of the paper that were used during peer review. These are the versions that the peer review comments apply to.

The versions of the paper that follow are the versions that were submitted during the peer review process. They are not the versions of record or the final published versions. They should not be cited or distributed as the published version of this manuscript.

BMJ Open is an open access journal and the full, final, typeset and author-corrected version of record of the manuscript is available on our site with no access controls, subscription charges or pay-per-view fees (http://bmjopen.bmj.com).

If you have any questions on BMJ Open's open peer review process please email info.bmjopen@bmj.com

BMJ Open

Longitudinal symptom profile of palliative care patients receiving a nurse-led end of life (PEACH) Program to support preference to die at home

Journal:	*BMJ Open*
Manuscript ID	bmjopen-2021-058448
Article Type:	Original research
Date Submitted by the Author:	05-Nov-2022
Complete List of Authors:	Agar, Meera; South Western Sydney Local Health District<br>Xuan, Wei; Ingham Institute of Applied Medical Research<br>Lee, Jessica; Sydney Local Health District<br>Barclay, Gregory; Illawarra Shoalhaven Local Health District<br>Oloffs, Alan; Nepean Blue Mountains Local Health District<br>Jobburn, Kim; South Western Sydney Local Health District<br>Harlum, Janeane; South Western Sydney Local Health District<br>Maurya, Nutan; South Western Sydney Local Health District<br>Chow, Josephine; South Western Sydney Local Health District, Clinical Innovation & Business Unit
Keywords:	PALLIATIVE CARE, Adult palliative care < PALLIATIVE CARE, GENERAL MEDICINE (see Internal Medicine)

SCHOLARONE™
Manuscripts

I, the Submitting Author has the right to grant and does grant on behalf of all authors of the Work (as defined in the below author licence), an exclusive licence and/or a non-exclusive licence for contributions from authors who are: i) UK Crown employees; ii) where BMJ has agreed a CC-BY licence shall apply, and/or iii) in accordance with the terms applicable for US Federal Government officers or employees acting as part of their official duties; on a worldwide, perpetual, irrevocable, royalty-free basis to BMJ Publishing Group Ltd ("BMJ") its licensees and where the relevant Journal is co-owned by BMJ to the co-owners of the Journal, to publish the Work in this journal and any other BMJ products and to exploit all rights, as set out in our licence.

The Submitting Author accepts and understands that any supply made under these terms is made by BMJ to the Submitting Author unless you are acting as an employee on behalf of your employer or a postgraduate student of an affiliated institution which is paying any applicable article publishing charge ("APC") for Open Access articles. Where the Submitting Author wishes to make the Work available on an Open Access basis (and intends to pay the relevant APC), the terms of reuse of such Open Access shall be governed by a Creative Commons licence – details of these licences and which Creative Commons licence will apply to this Work are set out in our licence referred to above.

Other than as permitted in any relevant BMJ Author's Self Archiving Policies, I confirm this Work has not been accepted for publication elsewhere, is not being considered for publication elsewhere and does not duplicate material already published. I confirm all authors consent to publication of this Work and authorise the granting of this licence.

For peer review only

Manuscript Title:

Longitudinal symptom profile of palliative care patients receiving a nurse-led end of life (PEACH) Program to support preference to die at home

Corresponding author (Full name, postal address and e-mail):

Professor Josephine Sau Fan Chow

Address: 1 Campbell Street, Liverpool NSW 1871

E mail: Josephine.Chow@health.nsw.gov.au

Full name, department, institution, city and country of all co-authors:

Meera R Agar,[1,2] Wei Xuan,[3] Jessica Lee,[4, 5] Greg Barclay,[6] Alan Oloffs,[7] Kim Jobburn,[8] Janeane Harlum,[2] Nutan Maurya,[8] Josephine Sau Fan Chow[8, 9, 10, 11] On behalf of the PEACH Research and Evaluation Working Group

[1]Centre for Improving Palliative, Aged and Chronic Care through Clinical Research and Translation (IMPACCT) & Faculty of Health, University of Technology Sydney, Sydney, New South Wales, Australia

[2]Department of Palliative Care, Cancer Services South Western Sydney Local Health District, Liverpool, Sydney, New South Wales, Australia.

[3]South Western Sydney Clinical School, University of New South Wales, Liverpool, Sydney, New South Wales, Australia.

[4]Concord Centre for Palliative Care, Concord Repatriation General Hospital, Sydney, New South Wales, Australia

[5]Concord Clinical School, University of Sydney, New South Wales, Australia.

For peer review only

[6]Palliative care services, Illawarra Shoalhaven Local Health District, New South Wales, Australia.

[7]Nepean Cancer Centre, Nepean Hospital, Nepean Blue Mountains Local Health District, Penrith, New South Wales, Australia.

[8]Clinical Innovation and Business Unit, South Western Sydney Local Health District, Liverpool, Sydney, New South Wales, Australia.

[9]South Western Sydney Nursing & Midwifery Research Alliance, Ingham Institute Applied Medical Research, Liverpool, Sydney, New South Wales, Australia

[10]Faculty of Medicine, University of New South Wales, Sydney, New South Wales, Australia

[11]NICM Health Research Institute, Western Sydney University, New South Wales, Australia.

Word count (excluding title page, abstract, references, figures and tables): 3135

For peer review only

ABSTRACT

Objectives: Tailored models of home-based palliative care aimed to support death at home, should also ensure optimal symptom control. This study aimed to explore symptom occurrence and distress over time in PEACH model of care recipients.

Setting: This was a prospective cohort study.

Participants: Participants were consecutive recipients of the PEACH rapid response nurse-led model of care in metropolitan Sydney (Dec 2013-Jan 2017) who were in the last weeks of life with a terminal or deteriorating phase of illness and had a preference to die at home.

Outcome Measures: De-identified data including sociodemographic and clinical characteristics, and symptom distress scores (Symptom Assessment Score) were collected at each clinical visit. Descriptive statistics and forward selection logistic regression analysis were used to explore influence of symptom distress levels on mode of separation across 4 symptom distress level categories.

Results: 1,754 consecutive clients received a PEACH package (Mean age 70 yrs, 55% male). 75.7% (n=1327) had a home death, 13.5% (n=237) were admitted and 10.8% (n=190) were still alive and residing at home when the package ceased. Mean symptom distress scores improved from baseline to final scores in the three groups ($P<0.0001$). The frequency of no symptom distress score (0) category was higher in the home death group. Higher scores for nausea, fatigue, insomnia, and bowel problems were independent predictors of who was admitted.

For peer review only

Conclusion: Tailored home-based palliative care models to meet preference to die at home, achieve this whilst maintaining symptom control. A focus on particular symptoms may further optimise these models of care.

What is already known about the topic?

- Home-based palliative care services increase the odds of dying at home with adequate symptom control but there is room for improvement.

What this paper adds

- The study demonstrates that a rapid response tailored home-based palliative care model of care allows the majority of people who have a preference to die at home to meet this preference.
- This model of care supports palliative care patients to meet their preference to die at home whilst maintaining good symptom control.
- Higher symptom distress scores for nausea, fatigue, insomnia, and bowel problems are independent predictors of those who had a preference to die at home who end up admitted to hospital.
- Fatigue remains a symptom which causes moderate symptom distress.

Implications for practice, theory or policy

- High symptom distress is an important indicator for increasing clinical support, and clinical models which are nimble to respond.
- The management of fatigue or support to minimise its distress remains an priority for end of life care.

For peer review only

BACKGROUND

Symptom burden is one of the most distressing aspects of the end of life (EOL) experience for people with a palliative diagnosis and for their families and loved ones[1 2] The symptoms which are prevalent near the EOL are well documented and include breathlessness and respiratory secretions, pain, fatigue, anorexia, and gastrointestinal symptoms.[2 3]

Home-based Palliative care services have been expanding worldwide, and many people at the EOL prefer to be care for and in many cases also die at home.[4-6] Community palliative care services have been demonstrated to improve patients and caregivers' experience at EOL through better symptom control, coordination of care and improved communication between professionals, patient and family and increase the odds of home deaths.[7 8] A recent review also suggests the strongest evidence for cost effectiveness relates to home-based interventions through both decreased healthcare costs and resource use and improvements in patient and caregiver outcomes[9] However they also indicated that there is still room to improve home palliative care services and increase benefits to patients, inclusive of those who wish to die at home.[8-10] A study of 25,679 patients who died under the care of a hospital or home-based palliative care team in Australia found that though over 85% of all patients had no severe symptoms prior to death, patients at home had less improvement over all and some symptoms got worse.[11]

The PEACH (*Palliative Extended And Care at Home*) model of care (a rapid response nursing care package delivered in conjunction with existing palliative care community services was developed for those who had high or complex palliative care needs to support care at home. Evaluation demonstrated it achieved increased days at home compared to usual care, with the PEACH model of care costs offset by reduced costs due to lower inpatient care needs.[12 13]

For peer review only

The PEACH model of care was then further tailored to provide EOL care in the last week of life for those patients who had an expressed wish to die at home. A prospective cohort study found that the majority of PEACH recipients with a clear preference to die at home when the service was initiated were able to die at home.[14] Though preference for place of death was achieved it is also important to understand if symptom control was optimal for those who received this model of care.

This study aimed to evaluate symptom occurrence and level of symptom distress over time in recipients of the PEACH model of care, and explore the associations of symptom distress with mode of separation.

For peer review only

METHODS

Study Design

Prospective cohort study. Ethical approval was granted from South Western Sydney Local Health District Human Research Ethics Committee (LNR/15/LPOOL/591) and Silver Chain Human Research Ethics Committee (EC App 144).

Study Population

Patients registered with the specialist palliative care services in five Local Health Districts (LHDs) in metropolitan Sydney, were eligible to receive a PEACH package if they were assessed as likely to be in the last week of life (terminal or deteriorating phase of illness[15] and Australia-modified Karnofsky Performance Scale (AKPS) score ≤40[16], have burdensome symptoms and/or require increased level of support, and had a preference to die at home. Consecutive recipients of the PEACH package between December 2013 and January 2017 were included.

Patient and public involvement

Patients or the public were not involved in the design, or conduct, or reporting, or dissemination plans of our research

Study Setting

The PEACH Program commenced in 2013 funded by the New South Wales Government, Australia in the participating health districts to address the needs of the community with a palliative diagnosis to achieve a home death.[14] The PEACH Program is coordinated through one LHD in metropolitan Sydney, Australia to provide packages across five New South Wales metropolitan LHDs (South Western Sydney, Illawarra Shoalhaven, Nepean Blue Mountains,

For peer review only

Sydney and Western Sydney) with PEACH nursing services delivered by a non-government organisation (Silver Chain Group). Australia provides universal healthcare (Medicare), which may be supplemented by private health insurance.

Study intervention

The PEACH package is centrally coordinated through a hub which coordinates community care services including community palliative care. The package is in addition to existing services delivered by General Practitioners, Community Nurses and Specialist Palliative Care Teams. The extra services provided were by a PEACH assistant in nursing during day time to provide either personal care or some respite to the carer, and a PEACH registered nurse visit in the evening and overnight telephone or video support by an experienced palliative care nurse who were fully briefed of the patient's care plan.

Data collection and variables

Data was collected routinely for all consecutive PEACH package recipients until separation from the package. De-identified data were used from administrative and clinical information systems obtaining the prospectively collected standardized data requirements and clinical assessments which were a requirement at referral to the PEACH program and during delivery of the PEACH package. To ensure confidentiality and anonymity data was collected using an alpha-numeric code for each participant.

For peer review only

Symptom occurrence and symptom distress

Symptom distress was assessed using Symptom Assessment Scale (SAS), which measures the perceived distress from common symptoms experienced by people receiving palliative care. and is a valid and reliable patient reported outcome measure suitable for routine clinical care with patients requiring palliative and EOL.[17] The scale assessed eight symptom dimensions: pain, insomnia (difficulty sleeping), nausea, vomiting, bowel problems, appetite problems, breathing problems, and fatigue on a 11 point numerical scale with zero being no distress due to that symptom and 10 being the worst distress possible. Symptom distress was categorized into four levels based on the score for each symptom dimension (0, >0 - ≤3, >3 - ≤6 and >6 - ≤10).

Mode of separation

Mode of separation, which was defined as the status of the patient at the end of the PEACH package episode of care (discharge, transfer or death) and the place to which the person is transferred.[18] Three possible modes of separation were identified from the PEACH package, namely i) person died at home whilst still receiving a PEACH package, ii) was admitted to a hospital or inpatient palliative care unit or if iii) were discharged from the package (alive and no longer requiring PEACH).

Socio-demographic and clinical variables

At the time of referral to the PEACH program socio-demographic clinical data was collected including age, gender, caregiver relationship to client (spouse, child, grandchild, other relative, others), preference for place of death on referral (classified as home,

For peer review only - http://bmjopen.bmj.com/site/about/guidelines.xhtml

hospital/Palliative Care Unit, or undecided), diagnosis (malignant, non-malignant), Palliative Care Phase on referral, location of referral (hospital/Palliative Care Unit, community), language spoken (English, Some English, no English), and as a proxy measure of geographical distance to admitted hospital services Local Government Area (metropolitan, inner and outer regional, remote or very remote as defined by Australian Classification of Local Governments (ACLG) determined by the Australian Bureau of Statistics (ABS)).

Statistical analyses

Data was analysed using SAS software (Version 9.4). Clinical and demographic characteristics were summarised using frequency counts and percentages for categorical data and the mean and standard deviation (SD) for continuous variables.

Mean Symptom Assessment Scale Scores at baseline (on referral) and final (average of the two scores recorded prior to separation) were calculated and compared for each symptom by the frequency and four levels of symptom distress by mode of separation (PEACH recipients who died at home versus those who were admitted to hospital or Palliative Care Unit as mode of separation). Forward selection logistic regression analysis were used to explore influence of symptom distress levels on mode of separation across the four symptom distress level categories.

For peer review only

RESULTS

Recipients of the PEACH model of care

A total of 1,754 consecutive palliative care patients (Table 1) received a PEACH package across the 5 LHDs from December 2013 to January 2017. The full participant characteristics are described elsewhere[14] but in summary the majority had a cancer diagnosis, mean age was 70 years (0-106), and 55.4 % (n=978) of them were male. Most patients reported their preferred place of death to be home (89%, n=1561) with smaller numbers citing the hospital or Palliative Care Unit (3.8%, n=67), and 7.2% (n=126) of the clients were not sure about their preferences. Overall, 75.7% (n=1327) clients died at home, 13.5% (n=237) were admitted to hospital or PCU and 10.8% (N=190) were alive at the end of the episode of care from PEACH program.

Table 1: Socio-demographic characteristics at referral to the PEACH program by the three modes of separation

	Died at home	Admitted to Hospital of Palliative Care Unit	Alive and no longer requiring PEACH
**Age,**			
Mean (SD)	70.8 (14.7)	69.3 (14.2)	68.4 (18.0)
**Gender** (n, %)			
Female	605 (45.6)	96 (40.5)	83 (43.7)
Male	722 (54.4)	141 (59.5)	107 (56.3)
**Diagnostic category** (n, %)			
Cancer	1094 (82.5)	214 (90.3)	146 (76.8)
Non-cancer	232 (17.50)	23 (9.7)	44 (23.2)
**Referral source** (n, %)			
Community Health Centre	1014 (76.4)	174 (73.4)	130 (68.4)
Hospital/Palliative Care Unit – Ward/Others	313 (23.6)	63 (26.6)	60 (31.6)
**Language** (n, %)			
English	1126 (84.9)	207 (87.3)	160 (84.2)
Some English	77 (5.8)	13 (5.5)	8 (4.2)
Non-English speaking	124 (9.3)	17 (7.2)	22 (11.6)
**Preferred place of death** (n, %)			
Home	1229 (92.6)	181 (76.4)	151 (79.5)
Hospital/Palliative Care Unit	28 (2.1)	25 (10.6)	14 (7.4)

For peer review only

Undecided	70 (5.3)	31 (13.1)	25 (13.2)
**LGA category** (n, %)			
Major city	1013 (76.3)	195 (82.3)	154 (81.1)
Inner regional	288 (21.7)	39 (16.5)	35 (18.4)
Outer regional	26 (2.0)	3 (1.3)	1 (0.5)
**Carer relationship** (n, %)			
Spouse	657 (50.0)	150 (63.8)	97 (51.6)
Child/Grand Child	517 (39.4)	67 (28.5)	70 (37.2)
Others	96 (7.3)	11 (4.7)	16 (8.5)
Sibling	43 (3.3)	7 (3.0)	5 (2.7)

Symptom distress over time by mode of separation

In the group who died at home, the mean symptom distress scores all improved remaining in the mild symptom distress category, though fatigue showed the least improvement and was rated as moderate levels of symptom distress (Table 2). A similar pattern was seen for those who were admitted to a hospital or palliative care unit, or who were discharged from the PEACH package except for fatigue which worsened in both groups. The baseline and final scores were comparatively higher in the group who were admitted as their mode of separation than the other two groups.

Table 2: Baseline and final Symptom Assessment Scores (mean) by mode of separation

	**Died at home**		**Admitted to Hospital of Palliative Care Unit**		**Alive and no longer requiring PEACH**	
	**Baseline**	**Final**	**Baseline**	**Final**	**Baseline**	**Final**
**Insomnia**	1.42	0.69	1.60	1.24	1.36	0.73
**Appetite**	2.04	1.05	2.43	1.75	2.36	1.25
**Nausea**	1.29	0.18	1.39	0.51	1.42	0.52
**Bowel Problems**	1.45	0.79	1.58	1.35	1.33	0.76
**Breathing Problems**	2.06	2.00	1.96	1.35	2.10	1.09
**Fatigue**	5.02	4.98	4.87	6.06	5.03	5.36

For peer review only

**Pain score**	1.88	1.59	1.98	1.91	1.58	1.15
**Vomiting score**	0.29	0.12	0.44	0.30	0.44	0.19

When the symptom distress scores were compared for the three modes of separation, significant differences in the baseline score was observed by mode of separation for the appetite only (p=0.044), with a higher score in the group admitted (2.43±2.90) compared to those who died at home (2.04±2.74) and those who remained at home alive on discharge from PEACH package (2.36±2.76) (Table 3). Significant differences in the symptom distress scores prior to separation was observed for all symptoms by mode of separation (Table 3).

Table 3: Comparison of baseline and final symptom distress scores by mode of separation

	Died at home		Admitted to Hospital of Palliative Care Unit		Alive and no longer requiring PEACH		
**Baseline score**	**N=1586**		**N=303**		**N=214**		
	**Mean**	**SD**	**Mean**	**SD**	**Mean**	**SD**	**p-value**
Insomnia	1.42	2.11	1.60	2.23	1.36	1.84	0.346
Appetite	2.04	2.74	2.43	2.90	2.36	2.76	0.044
Nausea	1.29	2.42	1.39	2.36	1.42	2.34	0.648
Bowel Problems	1.45	2.21	1.58	2.43	1.33	2.15	0.473
Breathing Problems	2.06	2.56	1.96	2.53	2.10	2.57	0.810
Fatigue	5.02	3.34	4.87	3.09	5.03	3.07	0.749
Pain score	1.88	2.30	1.98	2.41	1.58	2.04	0.324
Vomiting score	0.29	1.08	0.44	1.44	0.44	1.13	0.124
**Final score**	**N=1327**		**N=237**		**N=190**		
	**Mean**	**SD**	**Mean**	**SD**	**Mean**	**SD**	**p-value**
Insomnia	0.69	1.69	1.24	2.21	0.73	1.58	<.0001
Appetite	1.05	2.58	1.75	2.55	1.25	2.09	0.0006
Nausea	0.18	0.87	0.51	1.49	0.52	1.46	<.0001

For peer review only

Bowel Problems	0.79	1.73	1.35	2.13	0.76	1.58	<.0001
Breathing Problems	2.00	2.41	1.35	2.29	1.09	1.76	<.0001
Fatigue	4.98	4.03	6.06	2.49	5.36	2.66	0.0002
Pain score	1.59	2.11	1.91	2.54	1.15	1.99	0.0018
Vomiting score	0.12	0.73	0.30	1.34	0.19	0.86	0.0112

The final symptom distress scores by the four categories (score =0, >0 - ≤3, >3 - ≤6 and >6 - ≤10) for each symptom for the two modes of separation (died at home, admitted) is outlined in Table 4. The frequency of no symptom distress score (0) category was higher in the group who died at home, as compared to the group who were admitted to hospital. The higher score category for nausea, fatigue, insomnia and bowel problems were associated with being admitted to hospital.

For peer review only

Table 4: Final symptom distress by category for those who died at home and those who were admitted

Symptom distress category (score range)		Frequency, N (%)		OR	95% CI
		Died at home	Admitted		
**Insomnia**		N=1321	N=237		
	=0	942 (87.1)	139 (12.9)		
	>0 - ≤3	266 (79.2)	70 (20.8)	1.78	1.30-2.45
	>3 - ≤6	96 (82.1)	21 (17.9)	1.48	0.90-2.46
	>6 - ≤10	17 (70.8)	7 (29.2)	2.79	1.14-6.85
**Appetite**		N=1271	N=235		
	=0	880 (89.6)	102 (10.4)		
	>0 - ≤3	224 (71.8)	88 (28.2)	3.39	2.46-4.67
	>3 - ≤6	106 (76.3)	33 (23.7)	2.69	1.73-4.18
	>6 - ≤10	61 (83.6)	12 (16.4)	1.70	0.88-3.26
**Nausea**		N=1318	N=237		
	=0	1182 (86.8)	180 (13.2)		
	>0 - ≤3	112 (72.3)	43 (27.7)	2.52	1.72-3.71
	>3 - ≤6	19 (65.5)	10 (34.5)	3.46	1.58-7.55
	>6 - ≤10	5 (55.6)	4 (44.4)	5.25	1.40-19.75
**Bowel problem**		N=1322	N=237		
	=0	836 (88.3)	111 (11.7)		
	>0 - ≤3	370 (79.4)	96 (20.6)	1.95	1.45-2.64
	>3 - ≤6	97 (80.8)	23 (19.2)	1.79	1.09-2.93

For peer review only

	>6 - ≤10	19 (73.1)	7 (26.9)	2.78	1.14-6.75
**Breathing Problem**		N=1323	N=237		
	=0	504 (79.0)	134 (21.0)		
	>0 - ≤3	542 (90.2)	59 (9.8)	0.41	0.30-0.57
	>3 - ≤6	219 (85.6)	37 (14.5)	0.64	0.43-0.95
	>6 - ≤10	58 (89.2)	7 (10.8)	0.45	0.20-1.02
**Fatigue**		N=1311	N=237		
	=0	261 (98.5)	4 (1.5)		
	>0 - ≤3	86 (76.1)	27 (23.9)	20.48	6.97-60.20
	>3 - ≤6	314 (80.9)	74 (19.1)	15.38	5.55-42.62
	>6 - ≤10	650 (83.1)	132 (16.9)	13.25	4.85-36.20
**Pain**		N=1324	N=237		
	=0	469 (84.7)	85 (15.3)		
	>0 - ≤3	635 (85.7)	106 (14.3)	0.92	0.68-1.26
	>3 - ≤6	195 (85.2)	34 (14.9)	0.96	0.63-1.48
	>6 - ≤10	25 (67.6)	12 (32.4)	2.65	1.28-5.47
**Vomiting**		N=1320	N=237		
	=0	1246 (85.5)	212 (14.5)		
	>0 - ≤3	54 (74.0)	19 (26.0)	2.07	1.20-3.56
	>3 - ≤6	16 (84.2)	3 (15.8)	1.10	0.32-3.812
	>6 - ≤10	4 (57.1)	3 (42.9)	4.41	0.98-19.84

For peer review only

Association between symptom distress and mode of separation

On univariate analyses the higher the final symptom distress score (average of last two scores recorded prior to separation), the higher the odds of patient being admitted to hospital or palliative care unit as mode of separation, except for breathing problems which showed the reverse association (Table 5).

Table 5. Univariate analyses of symptom distress score by symptom for mode of separation (admitted to hospital or palliative care unit versus died at home)

Symptom score	OR	95%CI	p-value
Insomnia	1.13	1.048 - 1.221	0.0015
Appetite	1.11	1.048 - 1.168	0.0003
Nausea	1.31	1.178 - 1.465	<.0001
Bowel	1.13	1.054 - 1.218	0.0007
Breathing Problem	**0.88**	**0.812 - 0.945**	0.0006
Fatigue	1.09	1.041 - 1.144	0.0003
Pain	1.05	0.973 - 1.126	0.2175
Vomiting	1.21	1.063 - 1.377	0.0040

On multivariate analysis with forward selection higher final symptom distress scores for nausea, fatigue, insomnia and bowel problems were associated with being admitted to hospital (Table 6).

Table 6: Multivariate analyses (with forward selection) of symptom distress score by symptom for mode of separation (admitted to hospital or palliative care unit versus died at home)

Symptom score	OR	95%CI	p-value
(1) Nausea	1.25	1.12 - 1.41	<.0001

For peer review only - http://bmjopen.bmj.com/site/about/guidelines.xhtml

(2) Breathing	**0.85**	**0.79 -0.92**	<.0001
(3) Fatigue	1.08	1.03 -1.14	0.003
(4) Insomnia	1.10	1.02 - 1.20	0.02
(5) Bowel	1.09	1.01 - 1.18	0.024

For peer review only

For peer review only - http://bmjopen.bmj.com/site/about/guidelines.xhtml

DISCUSSION

This study has demonstrated that a tailored model of care to support palliative care patients who have a preference to die at home, supports excellent symptom control with symptom distress of mild severity and improving over time regardless of mode of separation from the PEACH package, apart from fatigue. The frequency of a rating of no symptom distress was highest in the group who died at home. The higher symptom distress for nausea, fatigue, insomnia and bowel problems were associated with being admitted to hospital. Symptom distress due to breathing problems were less likely to be admitted to hospital possibly reflecting the breathing changes in the imminently dying person where moving location of care would be inappropriate.

These findings are in the context of our prior study[14] finding that the majority of PEACH package recipients who have a clear preference to die at home when the service is initiated, were able to achieve their goal to die at home. People who were undecided at referral or had a preference for admission when death was imminent were more likely to be admitted to hospital or a PCU than die at home. People with a non-cancer diagnosis or who were cared for by their child or grandchild or friend/relative as caregiver were less likely to be admitted to hospital/PCU as a mode of separation.

We found that higher symptom distress score increases the likelihood of patient being admitted to the hospital or palliative care unit. This mirrors the findings of other studies which have reported that patients were more likely to be hospitalized or die in the hospital if they had higher functional status or greater pain intensity (pain score ≥ 2)[19] or when symptoms such as pain and dyspnoea were present.[20] The reason that the patients sought admission to the hospital or palliative care unit could be due to concerns in managing symptoms at home

For peer review only - http://bmjopen.bmj.com/site/about/guidelines.xhtml

which might require a level of supervision and physical assistance beyond caregivers' coping capacity, or to seek clinical assessment and adjustment to management, with these decisions may have been supported by the clinicians involved in their care in some cases.

Studies demonstrate a significant decrease in the level of severe symptom distress after start of palliative care. [11 21] Notably Gill and colleagues[21] found no change in the rates of fatigue ascertained from monthly interviews of 665 decedents of community living older people, and symptom that we also found caused moderate symptom distress increasing over time. Eagar and colleagues evaluated data derived from the Australian Palliative Care Outcomes Collaborative and found for 25,679 patients who died under the care of a hospital or home based palliative care team symptom distress improved and over 85% of patients had no severe symptom distress (scores 8 -10) just prior to death.[11] Similarly they found high rates for severe symptom distress for fatigue with this occurring in 14.6% of those in hospital and 10% of those at home just before death.

There is limited literature exploring differences in severe symptom outcomes for palliative care patients receiving hospital care compared with those receiving care at home. Some studies report that symptom outcomes are better for hospital patients and patients at home have less improvement overall and some symptoms get worse. A retrospective cohort study of 359 advanced cancer patients reported that those with final place of care as home had the lowest pain and depression scores as compared to patients in the inpatient hospice group or the hospital group.[19] A study in China found enhanced intensity of home palliative care visits with two additional days per week and formulated standard operating procedures for dyspnoea could significantly reduce the rate of ED visits by 30.7% (P < 0.05) due to dyspnoea during the last 6 months of life.[22] Nausea and well-being scores were also significantly worse

For peer review only - http://bmjopen.bmj.com/site/about/guidelines.xhtml

in the inpatient hospice group, compared to the home group.[19] It is not clear if those who were hospitalised had access to specialist palliative care. In contrast, Eagar and colleagues found that palliative care patients under the palliative care teams in the hospital were 3.7 times less likely than those at home to have no severely distressing symptoms before death.[11] This suggests that supplementing specialist palliative care with bespoke packages for the last week of life can ensure severe distress from symptoms is negated.

Strengths and limitations

This study is one of the largest cohorts of a tailored model of care aimed at improving the rates of home death for those where it was their expressed preference. The symptom assessments were collected as part of routine clinical care using a reliable and valid measure designed to be integrated into routine clinical care. This was a prospective cohort study and there is no usual care comparator, and the model was delivered in the Australian health care context. The majority of the recipients had cancer.

In this study, only symptoms assessed by the Symptom Assessment Scale were included in the analysis, there is possibility that patients might have other prominent or bothersome symptoms which may be unique to a particular palliative diagnosis that could have resulted in separation from the program. As the data collection ceased at separation from the package, the ongoing or change in symptom distress until death for patients admitted to hospital was unknown, though it is likely that these patients died during that index admission.

Recommendations

When designing home based palliative care models aimed to support palliative care patients at home, the model needs to be tailored to particular needs, time points in the illness

For peer review only - http://bmjopen.bmj.com/site/about/guidelines.xhtml

trajectory and preferences and evaluated to ensure ongoing capacity to refine the model to optimise patient outcomes. Further studies need to include more participants with non-cancer diagnoses. There is opportunity to further intensify support and symptom management when symptom distress is not improving or increasing, and fatigue warrants specific attention.

Conclusion

This study findings suggest that not only does the PEACH model of care allow people with palliative diagnoses to meet their preference to die at home, it achieves this whilst maintaining symptom control. Response to increase in particular symptoms may further optimise these models of care.

Ethics Statement

Patient consent for publication

Not required.

Ethics Approval

Ethical approval was granted from South Western Sydney Local Health District Human Research Ethics Committee (LNR/15/LPOOL/591) and Silver Chain Human Research Ethics Committee (EC App 144).

For peer review only - http://bmjopen.bmj.com/site/about/guidelines.xhtml

For peer review only

Meera R Agar,[1,2] Wei Xuan,[3] Jessica Lee,[4, 5] Greg Barclay,[6] Alan Oloffs,[7] Kim Jobburn,[8] Janeane Harlum,[2] Nutan Maurya,[8] Josephine Sau Fan Chow[8]

Contributorship Statement

MA, JC, WX, NM developed the idea and methodology of the study, provided governance and oversight and draft manuscript. JC, KJ, JH helped conduct the study, reviewed draft manuscripts. JC and KJ provided support and guidance of staff. MA, WX, NM analysed and interpreted the data. JL, GB, AO provided advice about study planning. JC, MA, WX and NM drafted the protocol and manuscript. All authors approved the final version of this manuscript.

Competing interests

The authors have no financial or professional relationships which may pose a competing interest.

Funding

This research received no specific grant funding from any funding agency in the public, commercial, or not-for-profit sectors.

Data sharing statement

Data for this study can be provided upon request.

For peer review only - http://bmjopen.bmj.com/site/about/guidelines.xhtml

For peer review only

REFERENCES:

1. Baillie J, Anagnostou D, Sivell S, et al. Symptom management, nutrition and hydration at end-of-life: a qualitative exploration of patients', carers' and health professionals' experiences and further research questions. *BMC Palliative Care* 2018;17(1):60. doi: 10.1186/s12904-018-0314-4

2. Singer AE, Meeker D, Teno JM, et al. Symptom trends in the last year of life from 1998 to 2010: a cohort study. *Ann Intern Med* 2015;162(3):175-83. doi: 10.7326/M13-1609

3. Kehl KA, Kowalkowski JA. A systematic review of the prevalence of signs of impending death and symptoms in the last 2 weeks of life. *Am J Hosp Palliat Care* 2013;30(6):601-16. doi: 10.1177/1049909112468222 [published Online First: 20121212]

4. Hoare S, Morris ZS, Kelly MP, et al. Do Patients Want to Die at Home? A Systematic Review of the UK Literature, Focused on Missing Preferences for Place of Death. *PLoS One* 2015;10(11):e0142723. doi: 10.1371/journal.pone.0142723 [published Online First: 2015/11/12]

5. Nysæter TM, Olsson C, Sandsdalen T, et al. Preferences for home care to enable home death among adult patients with cancer in late palliative phase – a grounded theory study. *BMC Palliative Care* 2022;21(1):49. doi: 10.1186/s12904-022-00939-y

6. Cai J, Zhang L, Guerriere D, et al. Where Do Cancer Patients in Receipt of Home-Based Palliative Care Prefer to Die and What Are the Determinants of a Preference for a Home Death? *Int J Environ Res Public Health* 2020;18(1):235. doi: 10.3390/ijerph18010235

For peer review only

7. Higginson IJ, Sarmento VP, Calanzani N, et al. Dying at home--is it better: a narrative appraisal of the state of the science. *Palliat Med* 2013;27(10):918-24. doi: 10.1177/0269216313487940 [published Online First: 2013/05/24]

8. Shepperd S, Goncalves-Bradley DC, Straus SE, et al. Hospital at home: home-based end-of-life care. *Cochrane Database Syst Rev* 2021;3:CD009231. doi: 10.1002/14651858.CD009231.pub3 [published Online First: 20210316]

9. Adamson E, Cruickshank S. A 'good death' at home: community nurses helping to make it possible. *Br J Community Nurs* 2013;18(1):40-2. doi: 10.12968/bjcn.2013.18.Sup9.S40 [published Online First: 2013/01/10]

10. Gomes B, Calanzani N, Higginson IJ. Benefits and costs of home palliative care compared with usual care for patients with advanced illness and their family caregivers. *Jama* 2014;311(10):1060-1. doi: 10.1001/jama.2014.553 [published Online First: 2014/03/13]

11. Eagar K, Clapham SP, Allingham SF. Palliative care is effective: but hospital symptom outcomes superior. *BMJ Support Palliat Care* 2020;10(2):186-90. doi: 10.1136/bmjspcare-2018-001534 [published Online First: 20180831]

12. McCaffrey N, Agar M, Harlum J, et al. Is home-based palliative care cost-effective? An economic evaluation of the Palliative Care Extended Packages at Home (PEACH) pilot. *BMJ Support Palliat Care* 2013;3(4):431-5. doi: 10.1136/bmjspcare-2012-000361 [published Online First: 2014/06/21]

13. McCaffrey N, Agar M, Harlum J, et al. Better informing decision making with multiple outcomes cost-effectiveness analysis under uncertainty in cost-disutility space. *PLoS One*

For peer review only - http://bmjopen.bmj.com/site/about/guidelines.xhtml

For peer review only

2015;10(3):e0115544. doi: 10.1371/journal.pone.0115544 [published Online First: 2015/03/10]

14. Agar MR, Xuan W, Lee J, et al. Factors Associated With Mode of Separation for People With Palliative Diagnoses With Preference for Home Death Receiving Care From a Nurse-Led End of Life (Palliative Extended and Care at Home) Program. *Journal of Hospice & Palliative Nursing* 9000

15. Eagar K, Gordon R, Green J, et al. An Australian casemix classification for palliative care: lessons and policy implications of a national study. *Palliat Med* 2004;18(3):227-33. doi: 10.1191/0269216304pm876oa

16. Abernethy AP, Shelby-James T, Fazekas BS, et al. The Australia-modified Karnofsky Performance Status (AKPS) scale: a revised scale for contemporary palliative care clinical practice [ISRCTN81117481]. *BMC Palliat Care* 2005;4:7. doi: 10.1186/1472-684X-4-7 [published Online First: 20051112]

17. Daveson BA, Allingham SF, Clapham S, et al. The PCOC Symptom Assessment Scale (SAS): A valid measure for daily use at point of care and in palliative care programs. *PLoS One* 2021;16(3):e0247250. doi: 10.1371/journal.pone.0247250 [published Online First: 20210325]

18. Australian Institute of Health and Welfare (AIHW). Mode of Separation Definition, National health data dictionary version 16. 2012. Accessed June 2022. https://www.aihw.gov.au/getmedia/a90ff140-8abf-4e30-8861-da2ebf368d35/14306.pdf.aspx?inline=true. [

19. Tay RY, Choo RWK, Ong WY, et al. Predictors of the final place of care of patients with advanced cancer receiving integrated home-based palliative care: a retrospective cohort

For peer review only - http://bmjopen.bmj.com/site/about/guidelines.xhtml

study. *BMC Palliat Care* 2021;20(1):164. doi: 10.1186/s12904-021-00865-5 [published Online First: 20211018]

20. Okamoto Y, Fukui S, Yoshiuchi K, et al. Do Symptoms among Home Palliative Care Patients with Advanced Cancer Decide the Place of Death? Focusing on the Presence or Absence of Symptoms during Home Care. *J Palliat Med* 2016;19(5):488-95. doi: 10.1089/jpm.2015.0184 [published Online First: 2016/02/06]

21. Gill TM, Gahbauer EA, Leo-Summers L, et al. Trends in Restricting Symptoms at the End of Life from 1998 to 2019: A Cohort Study of Older Persons. *J Am Geriatr Soc* 2021;69(2):450-58. doi: 10.1111/jgs.16871 [published Online First: 20201103]

22. Hsu H-S, Wu T-H, Lin C-Y, et al. Enhanced home palliative care could reduce emergency department visits due to non-organic dyspnea among cancer patients: a retrospective cohort study. *BMC Palliative Care* 2021;20(1):42. doi: 10.1186/s12904-021-00713-6

For peer review only

CONSORT 2010 checklist of information to include when reporting a randomised trial*

Section/Topic	Item No	Checklist item	Reported on page No
**Title and abstract**			
	1a	Identification as a randomised trial in the title	N/A
	1b	Structured summary of trial design, methods, results, and conclusions (for specific guidance see CONSORT for abstracts)	Abstract
**Introduction**			
Background and objectives	2a	Scientific background and explanation of rationale	2-3
	2b	Specific objectives or hypotheses	3
**Methods**			
Trial design	3a	Description of trial design (such as parallel, factorial) including allocation ratio	3
	3b	Important changes to methods after trial commencement (such as eligibility criteria), with reasons	N/A
Participants	4a	Eligibility criteria for participants	3
	4b	Settings and locations where the data were collected	4
Interventions	5	The interventions for each group with sufficient details to allow replication, including how and when they were actually administered	4
Outcomes	6a	Completely defined pre-specified primary and secondary outcome measures, including how and when they were assessed	4-5
	6b	Any changes to trial outcomes after the trial commenced, with reasons	N/A
Sample size	7a	How sample size was determined	N/A
	7b	When applicable, explanation of any interim analyses and stopping guidelines	N/A
Randomisation:			
Sequence generation	8a	Method used to generate the random allocation sequence	N/A
	8b	Type of randomisation; details of any restriction (such as blocking and block size)	N/A
Allocation concealment mechanism	9	Mechanism used to implement the random allocation sequence (such as sequentially numbered containers), describing any steps taken to conceal the sequence until interventions were assigned	N/A
Implementation	10	Who generated the random allocation sequence, who enrolled participants, and who assigned participants to interventions	N/A
Blinding	11a	If done, who was blinded after assignment to interventions (for example, participants, care providers, those	N/A

For peer review only

For peer review only - http://bmjopen.bmj.com/site/about/guidelines.xhtml

		assessing outcomes) and how	
	11b	If relevant, description of the similarity of interventions	N/A
Statistical methods	12a	Statistical methods used to compare groups for primary and secondary outcomes	6
	12b	Methods for additional analyses, such as subgroup analyses and adjusted analyses	N/A
**Results**			
Participant flow (a diagram is strongly recommended)	13a	For each group, the numbers of participants who were randomly assigned, received intended treatment, and were analysed for the primary outcome	N/A
	13b	For each group, losses and exclusions after randomisation, together with reasons	N/A
Recruitment	14a	Dates defining the periods of recruitment and follow-up	6
	14b	Why the trial ended or was stopped	N/A
Baseline data	15	A table showing baseline demographic and clinical characteristics for each group	7-8
Numbers analysed	16	For each group, number of participants (denominator) included in each analysis and whether the analysis was by original assigned groups	7-8
Outcomes and estimation	17a	For each primary and secondary outcome, results for each group, and the estimated effect size and its precision (such as 95% confidence interval)	9-11
	17b	For binary outcomes, presentation of both absolute and relative effect sizes is recommended	N/A
Ancillary analyses	18	Results of any other analyses performed, including subgroup analyses and adjusted analyses, distinguishing pre-specified from exploratory	9-11
Harms	19	All important harms or unintended effects in each group (for specific guidance see CONSORT for harms)	N/A
**Discussion**			
Limitations	20	Trial limitations, addressing sources of potential bias, imprecision, and, if relevant, multiplicity of analyses	15
Generalisability	21	Generalisability (external validity, applicability) of the trial findings	12-15
Interpretation	22	Interpretation consistent with results, balancing benefits and harms, and considering other relevant evidence	12-15
**Other information**			
Registration	23	Registration number and name of trial registry	N/A
Protocol	24	Where the full trial protocol can be accessed, if available	N/A
Funding	25	Sources of funding and other support (such as supply of drugs), role of funders	17

*We strongly recommend reading this statement in conjunction with the CONSORT 2010 Explanation and Elaboration for important clarifications on all the items. If relevant, we also recommend reading CONSORT extensions for cluster randomised trials, non-inferiority and equivalence trials, non-pharmacological treatments, herbal interventions, and pragmatic trials. Additional extensions are forthcoming: for those and for up to date references relevant to this checklist, see www.consort-statement.org.

For peer review only - http://bmjopen.bmj.com/site/about/guidelines.xhtml

For peer review only

BMJ Open

Longitudinal symptom profile of palliative care patients receiving a nurse-led end of life (PEACH) Program to support preference to die at home

Journal:	*BMJ Open*
Manuscript ID	bmjopen-2021-058448.R1
Article Type:	Original research
Date Submitted by the Author:	28-Sep-2023
Complete List of Authors:	Agar, Meera; South Western Sydney Local Health District<br>Xuan, Wei; Ingham Institute of Applied Medical Research<br>Lee, Jessica; Sydney Local Health District<br>Barclay, Gregory; Illawarra Shoalhaven Local Health District<br>Oloffs, Alan; Nepean Blue Mountains Local Health District<br>Jobburn, Kim; South Western Sydney Local Health District<br>Harlum, Janeane; South Western Sydney Local Health District<br>Maurya, Nutan; South Western Sydney Local Health District<br>Chow, Josephine; South Western Sydney Local Health District, Clinical Innovation & Business Unit
**Primary Subject Heading**:	Palliative care
Secondary Subject Heading:	Nursing, Palliative care
Keywords:	PALLIATIVE CARE, Adult palliative care < PALLIATIVE CARE, GENERAL MEDICINE (see Internal Medicine)

SCHOLARONE™
Manuscripts

I, the Submitting Author has the right to grant and does grant on behalf of all authors of the Work (as defined in the below author licence), an exclusive licence and/or a non-exclusive licence for contributions from authors who are: i) UK Crown employees; ii) where BMJ has agreed a CC-BY licence shall apply, and/or iii) in accordance with the terms applicable for US Federal Government officers or employees acting as part of their official duties; on a worldwide, perpetual, irrevocable, royalty-free basis to BMJ Publishing Group Ltd ("BMJ") its licensees and where the relevant Journal is co-owned by BMJ to the co-owners of the Journal, to publish the Work in this journal and any other BMJ products and to exploit all rights, as set out in our licence.

The Submitting Author accepts and understands that any supply made under these terms is made by BMJ to the Submitting Author unless you are acting as an employee on behalf of your employer or a postgraduate student of an affiliated institution which is paying any applicable article publishing charge ("APC") for Open Access articles. Where the Submitting Author wishes to make the Work available on an Open Access basis (and intends to pay the relevant APC), the terms of reuse of such Open Access shall be governed by a Creative Commons licence – details of these licences and which Creative Commons licence will apply to this Work are set out in our licence referred to above.

Other than as permitted in any relevant BMJ Author's Self Archiving Policies, I confirm this Work has not been accepted for publication elsewhere, is not being considered for publication elsewhere and does not duplicate material already published. I confirm all authors consent to publication of this Work and authorise the granting of this licence.

For peer review only

Manuscript Title:

Longitudinal symptom profile of palliative care patients receiving a nurse-led end of life (PEACH) Program to support preference to die at home

Corresponding author (Full name, postal address and e-mail):

Professor Josephine Sau Fan Chow

Address: 1 Campbell Street, Liverpool NSW 1871

E mail: Josephine.Chow@health.nsw.gov.au

Full name, department, institution, city and country of all co-authors:

Meera R Agar,[1,2] Wei Xuan,[3] Jessica Lee,[4, 5] Greg Barclay,[6] Alan Oloffs,[7] Kim Jobburn,[8] Janeane Harlum,[2] Nutan Maurya,[8] Josephine Sau Fan Chow[8, 9, 10, 11] On behalf of the PEACH Research and Evaluation Working Group

[1]Centre for Improving Palliative, Aged and Chronic Care through Clinical Research and Translation (IMPACCT) & Faculty of Health, University of Technology Sydney, Sydney, New South Wales, Australia

[2]Department of Palliative Care, Cancer Services South Western Sydney Local Health District, Liverpool, Sydney, New South Wales, Australia.

[3]South Western Sydney Clinical School, University of New South Wales, Liverpool, Sydney, New South Wales, Australia.

[4]Concord Centre for Palliative Care, Concord Repatriation General Hospital, Sydney, New South Wales, Australia

For peer review only - http://bmjopen.bmj.com/site/about/guidelines.xhtml

[5]Concord Clinical School, University of Sydney, New South Wales, Australia.

[6]Palliative care services, Illawarra Shoalhaven Local Health District, New South Wales, Australia.

[7]Nepean Cancer Centre, Nepean Hospital, Nepean Blue Mountains Local Health District, Penrith, New South Wales, Australia.

[8]Clinical Innovation and Business Unit, South Western Sydney Local Health District, Liverpool, Sydney, New South Wales, Australia.

[9]South Western Sydney Nursing & Midwifery Research Alliance, Ingham Institute Applied Medical Research, Liverpool, Sydney, New South Wales, Australia

[10]Faculty of Medicine, University of New South Wales, Sydney, New South Wales, Australia

[11]NICM Health Research Institute, Western Sydney University, New South Wales, Australia.

For peer review only

For peer review only - http://bmjopen.bmj.com/site/about/guidelines.xhtml

ABSTRACT

Objectives: Tailored models of home-based palliative care aimed to support death at home, should also ensure optimal symptom control. This study aimed to explore symptom occurrence and distress over time in Palliative Extended And Care at Home (PEACH) model of care recipients.

Design: This was a prospective cohort study

:.

Setting and Participants: Participants were consecutive recipients of the PEACH rapid response nurse-led model of care in metropolitan Sydney (Dec 2013-Jan 2017) who were in the last weeks of life with a terminal or deteriorating phase of illness and had a preference to be cared or die at home.

Outcome Measures: De-identified data including sociodemographic and clinical characteristics, and symptom distress scores (Symptom Assessment Score) were collected at each clinical visit. Descriptive statistics and forward selection logistic regression analysis were used to explore influence of symptom distress levels on mode of separation [(1) died at home while still receiving a PEACH package, (2) admitted to a hospital or an inpatient palliative care unit or (3) discharged from the package (alive and no longer requiring PEACH)] across 4 symptom distress level categories.

Results: 1,754 consecutive clients received a PEACH package (Mean age 70 years, 55% male). 75.7% (n=1327) had a home death, 13.5% (n=237) were admitted and 10.8% (n=190) were still alive and residing at home when the package ceased. Mean symptom distress scores improved from baseline to final scores in the three groups (P<0.0001). The frequency of no

For peer review only - http://bmjopen.bmj.com/site/about/guidelines.xhtml

For peer review only

symptom distress score (0) category was higher in the home death group. Higher scores for nausea, fatigue, insomnia, and bowel problems were independent predictors of who was admitted.

Conclusion: Tailored home-based palliative care models to meet preference to die at home, achieve this whilst maintaining symptom control. A focus on particular symptoms may further optimise these models of care.

Strengths & limitations of this study

- This study is one of the largest cohorts of a tailored model of care aimed at improving the rates of home death for those where it was their expressed preference.
- The symptom assessments were collected as part of routine clinical care using a reliable and valid measure designed to be integrated into routine clinical care.
- Only symptoms assessed by the Symptom Assessment Scale were included in the analysis.

For peer review only

For peer review only - http://bmjopen.bmj.com/site/about/guidelines.xhtml

BACKGROUND

Symptom burden is one of the most distressing aspects of the end of life (EOL) experience for people with a palliative diagnosis and for their families and loved ones[1, 2]. The symptoms which are prevalent near the EOL are well documented and include breathlessness and respiratory secretions, pain, fatigue, anorexia, and gastrointestinal symptoms[2, 3].

Home-based Palliative care services have been expanding worldwide, and many people at the EOL prefer to be care for and in many cases also die at home[4-6]. Community palliative care services have been demonstrated to improve patients and caregivers' experience at EOL through better symptom control, coordination of care and improved communication between professionals, patient and family and increase the odds of home deaths[7, 8]. A recent review also suggests the strongest evidence for cost effectiveness relates to home-based interventions through both decreased healthcare costs and resource use and improvements in patient and caregiver outcomes[9] However they also indicated that there is still room to improve home palliative care services and increase benefits to patients, inclusive of those who wish to die at home[8-10]. A study of 25,679 patients who died under the care of a hospital or home-based palliative care team in Australia found that though over 85% of all patients had no severe symptoms prior to death, patients at home had less improvement over all and some symptoms got worse[11].

The PEACH (*Palliative Extended And Care at Home*) model of care (a rapid response nursing care package delivered in conjunction with existing palliative care community services was developed for those who had high or complex palliative care needs to support care at home. Evaluation demonstrated it achieved increased days at home compared to usual care, with the PEACH model of care costs offset by reduced costs due to lower inpatient care needs[12,

For peer review only - http://bmjopen.bmj.com/site/about/guidelines.xhtml

13]. The PEACH model of care was then further tailored to provide EOL care in the last week of life for those patients who had an expressed wish to die at home. A prospective cohort study found that the majority of PEACH recipients with a clear preference to die at home when the service was initiated were able to die at home[14]. Though preference for place of death was achieved it is also important to understand if symptom control was optimal for those who received this model of care.

This study aimed to evaluate symptom occurrence and level of symptom distress over time in recipients of the PEACH model of care, and explore the associations of symptom distress with mode of separation.

For peer review only

For peer review only - http://bmjopen.bmj.com/site/about/guidelines.xhtml

METHODS

Study Design

Prospective cohort study. Ethical approval was granted from South Western Sydney Local Health District Human Research Ethics Committee (LNR/15/LPOOL/591) and Silver Chain Human Research Ethics Committee (EC App 144).

Study Population

Patients registered with the specialist palliative care services in five Local Health Districts (LHDs) in metropolitan Sydney, were eligible to receive a PEACH package if they were assessed as likely to be in the last week of life (terminal or deteriorating phase of illness[15] and Australia-modified Karnofsky Performance Scale (AKPS) score ≤40[16], have burdensome symptoms and/or require increased level of support, and had a preference to die at home. Consecutive recipients of the PEACH package between December 2013 and January 2017 were included.

Patient and public involvement

Patients or the public were not involved in the design, or conduct, or reporting, or dissemination plans of our research.

Study Setting

The PEACH Program is coordinated through one LHD in metropolitan Sydney, Australia to provide packages across five New South Wales metropolitan LHDs (South Western Sydney, Illawarra Shoalhaven, Nepean Blue Mountains, Sydney and Western Sydney) with PEACH nursing services delivered in partnership with a non-government organisation (Silver Chain

For peer review only - http://bmjopen.bmj.com/site/about/guidelines.xhtml

Group). Australia provides universal healthcare (Medicare), which may be supplemented by private health insurance.

Study intervention

The PEACH Program commenced in 2013 funded by the New South Wales Government, Australia in the participating health districts to address the needs of the community with a palliative diagnosis to facilitate a chosen home death while maintaining quality in the EOL care[14]. PEACH enables clients in their deteriorating and/or terminal phase to die at home or to stay at home as long as possible as per their wishes through provision of intensive, rapid, flexible, individualised nurse -led care and coordination for EOL care (last 7 days or can be extended) while maintaining satisfactory symptoms control and function at home.

Patients who were undecided about their preferred place of death but expressed an interest in attempting to stay home for EOL care or who wished to remain at home and transfer to hospital when death was imminent were also provided with a PEACH package. It was recognised for some clients there are a number of factors such as confidence in ability to die at home by either the patient and/or carer as well as cultural, religious, education or family demographics such as younger children in the home, impacted on preference for EOL care, i.e. undecided or transfer when death was imminent. Achieving end of life care preference was considered as one of the main objectives of the Program.

The PEACH package is centrally coordinated through a hub which coordinates community care services including community palliative care. The package provides some services in addition to existing services delivered by General Practitioners, Community Nurses and Specialist Palliative Care Teams. The additional services were provided by a PEACH assistant in nursing

For peer review only - http://bmjopen.bmj.com/site/about/guidelines.xhtml

during day time to provide either personal care or some respite to the carer, and a PEACH registered nurse visit in the evening and overnight telephone or video support by an experienced palliative care nurse who were fully briefed of the patient's care plan.

Data collection and variables

Data was collected routinely for all consecutive PEACH package recipients until separation from the package. De-identified data were used from administrative and clinical information systems obtaining the prospectively collected standardized data requirements and clinical assessments which were a requirement at referral to the PEACH program and during delivery of the PEACH package. To ensure confidentiality and anonymity data was collected using an alpha-numeric code for each participant.

Symptom occurrence and symptom distress

The median duration for patients on the program was 6 days. All patients as per the model of care were in receipt of daily clinical visits, which included a daily symptom score assessment until separation from the program. At times some clients/carers declined the daily visit and hence SAS would not be recorded for that day.

Symptom distress was assessed using Symptom Assessment Scale (SAS), which measures the perceived distress from common symptoms experienced by people receiving palliative care. and is a valid and reliable patient reported outcome measure suitable for routine clinical care with patients requiring palliative and EOL[17]. The scale assessed eight symptom dimensions: pain, insomnia (difficulty sleeping), nausea, vomiting, bowel problems, appetite problems, breathing problems, and fatigue on a 11 point numerical scale with zero being no distress due

For peer review only - http://bmjopen.bmj.com/site/about/guidelines.xhtml
For peer review only

to that symptom and 10 being the worst distress possible. Symptom distress was categorized into four levels based on the score for each symptom dimension (0, >0 - ≤3, >3 - ≤6 and >6 - ≤10).

Mode of separation

Mode of separation, which was defined as the status of the patient at the end of the PEACH package episode of care (discharge, transfer or death) and the place to which the person is transferred[18]. Three possible modes of separation were identified from the PEACH package, namely i) person died at home whilst still receiving a PEACH package, ii) was admitted to a hospital or inpatient palliative care unit or if iii) were discharged from the package (alive and no longer requiring PEACH).

Socio-demographic and clinical variables

At the time of referral to the PEACH program socio-demographic clinical data was collected including age, gender, caregiver relationship to client (spouse, child, grandchild, other relative, others), preference for place of death on referral (classified as home, hospital/Palliative Care Unit, or undecided), diagnosis (malignant, non-malignant), Palliative Care Phase on referral, location of referral (hospital/Palliative Care Unit, community), language spoken (English, Some English, no English), and as a proxy measure of geographical distance to admitted hospital services Local Government Area (metropolitan, inner and outer regional, remote or very remote as defined by Australian Classification of Local Governments (ACLG) determined by the Australian Bureau of Statistics (ABS)).

For peer review only

Statistical analyses

Data was analysed using SAS software (Version 9.4). Clinical and demographic characteristics were summarised using frequency counts and percentages for categorical data and the mean and standard deviation (SD) for continuous variables.

Mean Symptom Assessment Scale Scores at baseline (on referral) and final (average of the two scores recorded prior to separation) were calculated and compared for each symptom by the frequency and four levels of symptom distress by mode of separation (PEACH recipients who died at home versus those who were admitted to hospital or Palliative Care Unit as mode of separation). Forward selection logistic regression analysis were used to explore influence of symptom distress levels on mode of separation across the four symptom distress level categories.

For peer review only

RESULTS

Recipients of the PEACH model of care

A total of 1,754 consecutive palliative care patients (Table 1) received a PEACH package across the 5 LHDs from December 2013 to January 2017. The full participant characteristics are described elsewhere[14] but in summary the majority had a cancer diagnosis, mean age was 70 years (0-106), and 55.4 % (n=978) of them were male. Most patients reported their preferred place of death to be home (89%, n=1561) with smaller numbers citing the hospital or Palliative Care Unit (3.8%, n=67), and 7.2% (n=126) of the clients were not sure about their preferences. Overall, 75.7% (n=1327) clients died at home, 13.5% (n=237) were admitted to hospital or PCU and 10.8% (N=190) were alive at the end of the episode of care from PEACH program.

Table 1: Socio-demographic characteristics at referral to the PEACH program by the three modes of separation

	**Died at home (N=1327)**	**Admitted to Hospital OR Palliative Care Unit (N=237)**	**Alive and no longer requiring PEACH (N=190)**
**Age,**			
Mean (SD)	70.8 (14.7)	69.3 (14.2)	68.4 (18.0)
**Gender** (n, %)			
Female	605 (45.6)	96 (40.5)	83 (43.7)
Male	722 (54.4)	141 (59.5)	107 (56.3)
**Diagnostic category** (n, %)			
Cancer	1094 (82.5)	214 (90.3)	146 (76.8)
Non-cancer	232 (17.50)	23 (9.7)	44 (23.2)
**Referral source** (n, %)			
Community Health Centre	1014 (76.4)	174 (73.4)	130 (68.4)
Hospital/Palliative Care Unit – Ward/Others	313 (23.6)	63 (26.6)	60 (31.6)
**Language** (n, %)			
English	1126 (84.9)	207 (87.3)	160 (84.2)
Some English	77 (5.8)	13 (5.5)	8 (4.2)
Non-English speaking	124 (9.3)	17 (7.2)	22 (11.6)

For peer review only - http://bmjopen.bmj.com/site/about/guidelines.xhtml

**Preferred place of death** (n, %)			
Home	1229 (92.6)	181 (76.4)	151 (79.5)
Hospital/Palliative Care Unit	28 (2.1)	25 (10.6)	14 (7.4)
Undecided	70 (5.3)	31 (13.1)	25 (13.2)
**LGA category** (n, %)			
Major city	1013 (76.3)	195 (82.3)	154 (81.1)
Inner regional	288 (21.7)	39 (16.5)	35 (18.4)
Outer regional	26 (2.0)	3 (1.3)	1 (0.5)
**Carer relationship** (n, %)			
Spouse	657 (50.0)	150 (63.8)	97 (51.6)
Child/Grand Child	517 (39.4)	67 (28.5)	70 (37.2)
Others	96 (7.3)	11 (4.7)	16 (8.5)
Sibling	43 (3.3)	7 (3.0)	5 (2.7)

Symptom distress over time by mode of separation

In the group who died at home, the mean symptom distress scores all improved remaining in the mild symptom distress category, though fatigue showed the least improvement and was rated as moderate levels of symptom distress (Table 2). A similar pattern was seen for those who were admitted to a hospital or palliative care unit, or who were discharged from the PEACH package except for fatigue which worsened in both groups.

When the symptom distress scores were compared for the three modes of separation, no significant differences in the baseline score was observed by mode of separation . However a significant differences in the symptom distress scores prior to separation (final score) was observed for all symptoms by mode of separation (Table 2).

Table 2: Comparison of baseline and final symptom distress scores by mode of separation

	Died at home	Admitted to Hospital of	Alive and no longer

For peer review only - http://bmjopen.bmj.com/site/about/guidelines.xhtml

			Palliative Care Unit		requiring PEACH		
	N=1327		N=237		N=190		
**Baseline score**	**Mean**	**SD**	**Mean**	**SD**	**Mean**	**SD**	**p-value**
Insomnia	1.47	2.12	1.64	2.26	1.33	1.84	0.322
Appetite	2.16	2.74	2.37	2.79	2.45	2.78	0.297
Nausea	1.41	2.50	1.45	2.35	1.47	2.39	0.933
Bowel Problems	1.55	2.28	1.49	2.32	1.39	2.11	0.651
Breathing Problems	2.06	2.56	1.96	2.53	2.19	2.64	0.666
Fatigue	5.12	3.26	4.76	3.08	4.92	3.09	0.271
Pain score	1.89	2.26	1.84	2.36	1.60	2.07	0.477
Vomiting score	0.30	1.11	0.44	1.36	0.43	1.11	0.311
**Final score**	**Mean**	**SD**	**Mean**	**SD**	**Mean**	**SD**	**p-value**
Insomnia	0.69	1.69	1.24	2.21	0.73	1.58	<.0001
Appetite	1.05	2.58	1.75	2.55	1.25	2.09	0.0006
Nausea	0.18	0.87	0.51	1.49	0.52	1.46	<.0001
Bowel Problems	0.79	1.73	1.35	2.13	0.76	1.58	<.0001
Breathing Problems	2.00	2.41	1.35	2.29	1.09	1.76	<.0001
Fatigue	4.98	4.03	6.06	2.49	5.36	2.66	0.0002
Pain score	1.59	2.11	1.91	2.54	1.15	1.99	0.0018
Vomiting score	0.12	0.73	0.30	1.34	0.19	0.86	0.0112

For peer review only

The final symptom distress scores by the four categories (score =0, >0 - ≤3, >3 - ≤6 and >6 - ≤10) for each symptom for the two modes of separation (died at home, admitted) is outlined in Table 3. The other 3 categories (>0 - ≤3, >3 - ≤6 and >6 - ≤10) were compared to the =0 category. The frequency of no symptom distress score (0) category was higher in the group who died at home, as compared to the group who were admitted to hospital. The higher score category for nausea, fatigue, insomnia and bowel problems were associated with being admitted to hospital.

For peer review only - http://bmjopen.bmj.com/site/about/guidelines.xhtml

For peer review only

For peer review only - http://bmjopen.bmj.com/site/about/guidelines.xhtml

Table 3: Final symptom distress by category for those who died at home and those who were admitted

	Symptom distress category (score range)	Frequency, N (%)		P value
		Died at home	Admitted	
**Insomnia**		N=1321	N=237	
	=0	942 (87.1)	139 (12.9)	
	>0 - ≤3	266 (79.2)	70 (20.8)	0.0006
	>3 - ≤6	96 (82.1)	21 (17.9)	
	>6 - ≤10	17 (70.8)	7 (29.2)	
**Appetite**		N=1271	N=235	
	=0	880 (89.6)	102 (10.4)	
	>0 - ≤3	224 (71.8)	88 (28.2)	<.0001
	>3 - ≤6	106 (76.3)	33 (23.7)	
	>6 - ≤10	61 (83.6)	12 (16.4)	
**Nausea**		N=1318	N=237	
	=0	1182 (86.8)	180 (13.2)	
	>0 - ≤3	112 (72.3)	43 (27.7)	<.0001
	>3 - ≤6	19 (65.5)	10 (34.5)	
	>6 - ≤10	5 (55.6)	4 (44.4)	
**Bowel problem**		N=1322	N=237	
	=0	836 (88.3)	111 (11.7)	
	>0 - ≤3	370 (79.4)	96 (20.6)	<.0001
	>3 - ≤6	97 (80.8)	23 (19.2)	

For peer review only - http://bmjopen.bmj.com/site/about/guidelines.xhtml

	>6 - ≤10	19 (73.1)	7 (26.9)	
**Breathing Problem**		N=1323	N=237	
	=0	504 (79.0)	134 (21.0)	
	>0 - ≤3	542 (90.2)	59 (9.8)	<.0001
	>3 - ≤6	219 (85.6)	37 (14.5)	
	>6 - ≤10	58 (89.2)	7 (10.8)	
**Fatigue**		N=1311	N=237	
	=0	261 (98.5)	4 (1.5)	
	>0 - ≤3	86 (76.1)	27 (23.9)	<.0001
	>3 - ≤6	314 (80.9)	74 (19.1)	
	>6 - ≤10	650 (83.1)	132 (16.9)	
**Pain**		N=1324	N=237	
	=0	469 (84.7)	85 (15.3)	
	>0 - ≤3	635 (85.7)	106 (14.3)	0.0290
	>3 - ≤6	195 (85.2)	34 (14.9)	
	>6 - ≤10	25 (67.6)	12 (32.4)	
**Vomiting**		N=1320	N=237	
	=0	1246 (85.5)	212 (14.5)	
	>0 - ≤3	54 (74.0)	19 (26.0)	0.0103
	>3 - ≤6	16 (84.2)	3 (15.8)	
	>6 - ≤10	4 (57.1)	3 (42.9)	

For peer review only

Association between symptom distress and mode of separation

On univariate analyses the higher the final symptom distress score (average of last two scores recorded prior to separation), the higher the odds of patient being admitted to hospital or palliative care unit as mode of separation, except for breathing problems which showed the reverse association (Table 4).

Table 4. Univariate analyses of symptom distress score by symptom for mode of separation (admitted to hospital or palliative care unit versus died at home)

Symptom score	OR	95%CI	p-value
Insomnia	1.13	1.048 - 1.221	0.0015
Appetite	1.11	1.048 - 1.168	0.0003
Nausea	1.31	1.178 - 1.465	<.0001
Bowel	1.13	1.054 - 1.218	0.0007
Breathing Problem	**0.88**	**0.812 - 0.945**	0.0006
Fatigue	1.09	1.041 - 1.144	0.0003
Pain	1.05	0.973 - 1.126	0.2175
Vomiting	1.21	1.063 - 1.377	0.0040

On multivariate analysis with forward selection higher final symptom distress scores for nausea, fatigue, insomnia and bowel problems were associated with being admitted to hospital (Table 5).

Table 5: Multivariate analyses (with forward selection) of symptom distress score by symptom for mode of separation (admitted to hospital or palliative care unit versus died at home)

Symptom score	OR	95%CI	p-value
(1) Nausea	1.25	1.12 - 1.41	<.0001

For peer review only - http://bmjopen.bmj.com/site/about/guidelines.xhtml

(2) Breathing	**0.85**	**0.79 -0.92**	<.0001
(3) Fatigue	1.08	1.03 -1.14	0.003
(4) Insomnia	1.10	1.02 - 1.20	0.02
(5) Bowel	1.09	1.01 - 1.18	0.024

For peer review only

For peer review only - http://bmjopen.bmj.com/site/about/guidelines.xhtml

DISCUSSION

This study has demonstrated that a tailored model of care to support palliative care patients who have a preference to die at home, supports excellent symptom control with symptom distress of mild severity and improving over time regardless of mode of separation from the PEACH package, apart from fatigue. The frequency of a rating of no symptom distress was highest in the group who died at home. The higher symptom distress for nausea, fatigue, insomnia and bowel problems were associated with being admitted to hospital. Symptom distress due to breathing problems were less likely to be admitted to hospital possibly reflecting the breathing changes in the imminently dying person where moving location of care would be inappropriate.

These findings are in the context of our prior study [14] finding that the majority of PEACH package recipients who have a clear preference to die at home when the service is initiated, were able to achieve their goal to die at home. People who were undecided at referral or had a preference for admission when death was imminent were more likely to be admitted to hospital or a PCU than die at home. People with a non-cancer diagnosis or who were cared for by their child or grandchild or friend/relative as caregiver were less likely to be admitted to hospital/PCU as a mode of separation.

We found that higher symptom distress score increases the likelihood of patient being admitted to the hospital or palliative care unit. This mirrors the findings of other studies which have reported that patients were more likely to be hospitalized or die in the hospital if they had higher functional status or greater pain intensity (pain score ≥ 2)[19] or when symptoms such as pain and dyspnoea were present[20]. A recent home-based palliative care cohort study of adult cancer patients in Singapore showed that high symptom needs increased

For peer review only - http://bmjopen.bmj.com/site/about/guidelines.xhtml

For peer review only

incidence rate ratios of acute healthcare utilization[21]. The reason that the patients sought admission to the hospital or palliative care unit could be due to concerns in managing symptoms at home which might require a level of supervision and physical assistance beyond caregivers' coping capacity, or to seek clinical assessment and adjustment to management, with these decisions may have been supported by the clinicians involved in their care in some cases.

For peer review only

Studies demonstrate a significant decrease in the level of severe symptom distress after start of palliative care [11, 22]. [22] Notably Gill and colleagues[22] found no change in the rates of fatigue ascertained from monthly interviews of 665 decedents of community living older people, and symptom that we also found caused moderate symptom distress increasing over time. Eagar and colleagues evaluated data derived from the Australian Palliative Care Outcomes Collaborative and found for 25,679 patients who died under the care of a hospital or home based palliative care team symptom distress improved and over 85% of patients had no severe symptom distress (scores 8 -10) just prior to death[11].[1] Similarly they found high rates for severe symptom distress for fatigue with this occurring in 14.6% of those in hospital and 10% of those at home just before death.

There is limited literature exploring differences in severe symptom outcomes for palliative care patients receiving hospital care compared with those receiving care at home. Some studies report that symptom outcomes are better for hospital patients and patients at home have less improvement overall and some symptoms get worse. A retrospective cohort study of 359 advanced cancer patients reported that those with final place of care as home had the lowest pain and depression scores as compared to patients in the inpatient hospice group or the hospital group[19]. A study in China found enhanced intensity of home palliative care

For peer review only - http://bmjopen.bmj.com/site/about/guidelines.xhtml

visits with two additional days per week and formulated standard operating procedures for dyspnoea could significantly reduce the rate of ED visits by 30.7% ($P < 0.05$) due to dyspnoea during the last 6 months of life[23]. Nausea and well-being scores were also significantly worse in the inpatient hospice group, compared to the home group[19]. It is not clear if those who were hospitalised had access to specialist palliative care. In contrast, Eagar and colleagues found that palliative care patients under the palliative care teams in the hospital were 3.7 times less likely than those at home to have no severely distressing symptoms before death[11]. This suggests that supplementing specialist palliative care with bespoke packages for the last week of life can ensure severe distress from symptoms is negated.

Limitations

This was a prospective cohort study and there is no usual care comparator, and the model was delivered in the Australian health care context which may limit generalizability of this model elsewhere. The majority of the recipients had cancer.

In this study, only symptoms assessed by the Symptom Assessment Scale were included in the analysis, there is possibility that patients might have other prominent or bothersome symptoms which may be unique to a particular palliative diagnosis that could have resulted in separation from the program.

As the data collection ceased at separation from the package, the ongoing or change in symptom distress until death for patients admitted to hospital was unknown, though it is likely that these patients died during that index admission.

Recommendations

For peer review only - http://bmjopen.bmj.com/site/about/guidelines.xhtml

When designing home based palliative care models aimed to support palliative care patients at home, the model needs to be tailored to particular needs, time points in the illness trajectory and preferences and evaluated to ensure ongoing capacity to refine the model to optimise patient outcomes. Further studies need to include more participants with non-cancer diagnoses. There is opportunity to further intensify support and symptom management when symptom distress is not improving or increasing, and fatigue warrants specific attention.

Conclusion

This study findings suggest that not only does the PEACH model of care allow people with palliative diagnoses to meet their preference to die at home, it achieves this whilst maintaining symptom control. Response to increase in particular symptoms may further optimise these models of care.

Ethics Statement

Patient consent for publication

Not required.

Ethics Approval

For peer review only

Ethical approval was granted from South Western Sydney Local Health District Human Research Ethics Committee (LNR/15/LPOOL/591) and Silver Chain Human Research Ethics Committee (EC App 144).

Meera R Agar,[1,2] Wei Xuan,[3] Jessica Lee,[4, 5] Greg Barclay,[6] Alan Oloffs,[7] Kim Jobburn,[8] Janeane Harlum,[2] Nutan Maurya,[8] Josephine Sau Fan Chow[8]

Contributorship Statement

MA, JC, WX, NM developed the idea and methodology of the study, provided governance and oversight and draft manuscript. JC, KJ, JH helped conduct the study, reviewed draft manuscripts. JC and KJ provided support and guidance of staff. MA, WX, NM analysed and interpreted the data. JL, GB, AO provided advice about study planning. JC, MA, WX and NM drafted the protocol and manuscript. All authors approved the final version of this manuscript.

Competing interests

The authors have no financial or professional relationships which may pose a competing interest.

Funding

This research received no specific grant funding from any funding agency in the public, commercial, or not-for-profit sectors.

Data sharing statement

For peer review only - http://bmjopen.bmj.com/site/about/guidelines.xhtml

Data for this study can be provided upon request.

For peer review only

REFERENCES:

1. Baillie J, Anagnostou D, Sivell S, et al. Symptom management, nutrition and hydration at end-of-life: a qualitative exploration of patients', carers' and health professionals' experiences and further research questions. *BMC Palliative Care* 2018;17(1):60. doi: 10.1186/s12904-018-0314-4

2. Singer AE, Meeker D, Teno JM, et al. Symptom trends in the last year of life from 1998 to 2010: a cohort study. *Ann Intern Med* 2015;162(3):175-83. doi: 10.7326/M13-1609

3. Kehl KA, Kowalkowski JA. A systematic review of the prevalence of signs of impending death and symptoms in the last 2 weeks of life. *Am J Hosp Palliat Care* 2013;30(6):601-16. doi: 10.1177/1049909112468222 [published Online First: 20121212]

4. Hoare S, Morris ZS, Kelly MP, et al. Do Patients Want to Die at Home? A Systematic Review of the UK Literature, Focused on Missing Preferences for Place of Death. *PLoS One* 2015;10(11):e0142723. doi: 10.1371/journal.pone.0142723 [published Online First: 2015/11/12]

5. Nysæter TM, Olsson C, Sandsdalen T, et al. Preferences for home care to enable home death among adult patients with cancer in late palliative phase – a grounded theory study. *BMC Palliative Care* 2022;21(1):49. doi: 10.1186/s12904-022-00939-y

6. Cai J, Zhang L, Guerriere D, et al. Where Do Cancer Patients in Receipt of Home-Based Palliative Care Prefer to Die and What Are the Determinants of a Preference for a Home Death? *Int J Environ Res Public Health* 2020;18(1):235. doi: 10.3390/ijerph18010235

For peer review only

7. Higginson IJ, Sarmento VP, Calanzani N, et al. Dying at home--is it better: a narrative appraisal of the state of the science. *Palliat Med* 2013;27(10):918-24. doi: 10.1177/0269216313487940 [published Online First: 2013/05/24]

8. Shepperd S, Goncalves-Bradley DC, Straus SE, et al. Hospital at home: home-based end-of-life care. *Cochrane Database Syst Rev* 2021;3:CD009231. doi: 10.1002/14651858.CD009231.pub3 [published Online First: 20210316]

9. Adamson E, Cruickshank S. A 'good death' at home: community nurses helping to make it possible. *Br J Community Nurs* 2013;18(1):40-2. doi: 10.12968/bjcn.2013.18.Sup9.S40 [published Online First: 2013/01/10]

10. Gomes B, Calanzani N, Higginson IJ. Benefits and costs of home palliative care compared with usual care for patients with advanced illness and their family caregivers. *Jama* 2014;311(10):1060-1. doi: 10.1001/jama.2014.553 [published Online First: 2014/03/13]

11. Eagar K, Clapham SP, Allingham SF. Palliative care is effective: but hospital symptom outcomes superior. *BMJ Support Palliat Care* 2020;10(2):186-90. doi: 10.1136/bmjspcare-2018-001534 [published Online First: 20180831]

12. McCaffrey N, Agar M, Harlum J, et al. Is home-based palliative care cost-effective? An economic evaluation of the Palliative Care Extended Packages at Home (PEACH) pilot. *BMJ Support Palliat Care* 2013;3(4):431-5. doi: 10.1136/bmjspcare-2012-000361 [published Online First: 2014/06/21]

13. McCaffrey N, Agar M, Harlum J, et al. Better informing decision making with multiple outcomes cost-effectiveness analysis under uncertainty in cost-disutility space. *PLoS One*

For peer review only - http://bmjopen.bmj.com/site/about/guidelines.xhtml

2015;10(3):e0115544. doi: 10.1371/journal.pone.0115544 [published Online First: 2015/03/10]

14. Agar MR, Xuan W, Lee J, et al. Factors Associated With Mode of Separation for People With Palliative Diagnoses With Preference for Home Death Receiving Care From a Nurse-Led End of Life (Palliative Extended and Care at Home) Program. *Journal of Hospice & Palliative Nursing* 9000

15. Eagar K, Gordon R, Green J, et al. An Australian casemix classification for palliative care: lessons and policy implications of a national study. *Palliat Med* 2004;18(3):227-33. doi: 10.1191/0269216304pm876oa

16. Abernethy AP, Shelby-James T, Fazekas BS, et al. The Australia-modified Karnofsky Performance Status (AKPS) scale: a revised scale for contemporary palliative care clinical practice [ISRCTN81117481]. *BMC Palliat Care* 2005;4:7. doi: 10.1186/1472-684X-4-7 [published Online First: 20051112]

17. Daveson BA, Allingham SF, Clapham S, et al. The PCOC Symptom Assessment Scale (SAS): A valid measure for daily use at point of care and in palliative care programs. *PLoS One* 2021;16(3):e0247250. doi: 10.1371/journal.pone.0247250 [published Online First: 20210325]

18. Australian Institute of Health and Welfare (AIHW). Mode of Separation Definition, National health data dictionary version 16. 2012. Accessed June 2022. https://www.aihw.gov.au/getmedia/a90ff140-8abf-4e30-8861-da2ebf368d35/14306.pdf.aspx?inline=true.

19. Tay RY, Choo RWK, Ong WY, et al. Predictors of the final place of care of patients with advanced cancer receiving integrated home-based palliative care: a retrospective cohort

For peer review only - http://bmjopen.bmj.com/site/about/guidelines.xhtml

study. *BMC Palliat Care* 2021;20(1):164. doi: 10.1186/s12904-021-00865-5 [published Online First: 20211018]

20. Okamoto Y, Fukui S, Yoshiuchi K, et al. Do Symptoms among Home Palliative Care Patients with Advanced Cancer Decide the Place of Death? Focusing on the Presence or Absence of Symptoms during Home Care. *J Palliat Med* 2016;19(5):488-95. doi: 10.1089/jpm.2015.0184 [published Online First: 2016/02/06]

21. Zhuang Q, Chong PH, Ong WS, et al. Longitudinal patterns and predictors of healthcare utilization among cancer patients on home-based palliative care in Singapore: a group-based multi-trajectory analysis. *BMC Med*. 2022;20(1):313. Published 2022 Sep 22. doi:10.1186/s12916-022-02513-y

22. Gill TM, Gahbauer EA, Leo-Summers L, et al. Trends in Restricting Symptoms at the End of Life from 1998 to 2019: A Cohort Study of Older Persons. *J Am Geriatr Soc* 2021;69(2):450-58. doi: 10.1111/jgs.16871 [published Online First: 20201103]

23. Hsu H-S, Wu T-H, Lin C-Y, et al. Enhanced home palliative care could reduce emergency department visits due to non-organic dyspnea among cancer patients: a retrospective cohort study. *BMC Palliative Care* 2021;20(1):42. doi: 10.1186/s12904-021-00713-6

For peer review only - http://bmjopen.bmj.com/site/about/guidelines.xhtml

STROBE Statement—Checklist of items included in the manuscript

	Item No	Recommendation	Page
**Title and abstract**	1	(*a*) Indicate the study's design with a commonly used term in the title or the abstract	3
		b) Provide in the abstract an informative and balanced summary of what was done and what was found	3
**Introduction**			
Background/rationale	2	Explain the scientific background and rationale for the investigation being reported	6
Objectives	3	State specific objectives, including any prespecified hypotheses	7
**Methods**			
Study design	4	Present key elements of study design early in the paper	8
Setting	5	Describe the setting, locations, and relevant dates, including periods of recruitment, exposure, follow-up, and data collection	8-9
Participants	6	(*a*) Give the eligibility criteria, and the sources and methods of selection of participants. Describe methods of follow-up<br>(*b*) For matched studies, give matching criteria and number of exposed and unexposed	8
Variables	7	Clearly define all outcomes, exposures, predictors, potential confounders, and effect modifiers; give diagnostic criteria, if applicable	10
Data sources/measurement	8*	For each variable of interest, give sources of data and details of methods of assessment (measurement); describe comparability of assessment methods if there is more than one group	10-11
Bias	9	Describe any efforts to address potential sources of bias	-
Study size	10	Explain how the study size was arrived at	10
Quantitative variables	11	Explain how quantitative variables were handled in the analyses; if applicable, describe which groupings were chosen and why	10-11
Statistical methods	12	a) Describe all statistical methods, including those used to control for confounding<br>b) Describe any methods used to examine subgroups and interactions<br>c) Explain how missing data were addressed<br>d) Cohort study - if applicable, explain how loss to follow-up was addressed<br>e) Describe any sensitivity analyses	12
**Results**			
Participants	13*	a) Report numbers of individuals at each stage of study - e.g., numbers potentially eligible, examined for eligibility, confirmed eligible,	13

For peer review only

		included in the study, completing follow-up, and analyzed<br>b) Give reasons for nonparticipation at each stage.<br>c) Consider use of a flow diagram	
Descriptive data	14*	a) Give characteristics of study participants (e.g., demographic, clinical, social) and information on exposures and potential confounders<br>b) Indicate number of participants with missing data for each variable of interest<br>c) Summarize follow-up time (e.g., average and total amount)	13
Outcome data	15*	Report numbers of outcome events or summary measures over time	14-17
Main results	16	a) Give unadjusted estimates and, if applicable, confounder-adjusted estimates and their precision (e.g., 95% confidence interval); make clear which confounders were adjusted for and why they were included<br>b) Report category boundaries when continuous variables were categorized<br>c) If relevant, consider translating estimates of relative risk into absolute risk for a meaningful time period	18-23
Other analyses	17	Report other analyses done - e.g., analyses of subgroups and interactions, and sensitivity analyses	-
**Discussion**			
Key results	18	Summarize key results with reference to study objectives	24-26
Limitations	19	Discuss limitations of the study, taking into account sources of potential bias or imprecision; discuss both direction and magnitude of any potential bias	26
Interpretation	20	Give a cautious overall interpretation of results considering objectives, limitations, multiplicity of analyses, results from similar studies, and other relevant evidence	27
Generalizability	21	Discuss the generalizability (external validity) of the study results	26
**Other information**			
Funding	22	Give the source of funding and the role of the funders for the present study and, if applicable, for the original study on which the present article is based	29

For peer review only

BMJ Open

Longitudinal symptom profile of palliative care patients receiving a nurse-led end of life (PEACH) Program to support preference to die at home

Journal:	*BMJ Open*
Manuscript ID	bmjopen-2021-058448.R2
Article Type:	Original research
Date Submitted by the Author:	24-Nov-2023
Complete List of Authors:	Agar, Meera; South Western Sydney Local Health District<br>Xuan, Wei; Ingham Institute of Applied Medical Research<br>Lee, Jessica; Sydney Local Health District<br>Barclay, Gregory; Illawarra Shoalhaven Local Health District<br>Oloffs, Alan; Nepean Blue Mountains Local Health District<br>Jobburn, Kim; South Western Sydney Local Health District<br>Harlum, Janeane; South Western Sydney Local Health District<br>Maurya, Nutan; South Western Sydney Local Health District<br>Chow, Josephine; South Western Sydney Local Health District, Clinical Innovation & Business Unit
**Primary Subject Heading**:	Palliative care
Secondary Subject Heading:	Nursing, Palliative care
Keywords:	PALLIATIVE CARE, Adult palliative care < PALLIATIVE CARE, GENERAL MEDICINE (see Internal Medicine)

SCHOLARONE™
Manuscripts

I, the Submitting Author has the right to grant and does grant on behalf of all authors of the Work (as defined in the below author licence), an exclusive licence and/or a non-exclusive licence for contributions from authors who are: i) UK Crown employees; ii) where BMJ has agreed a CC-BY licence shall apply, and/or iii) in accordance with the terms applicable for US Federal Government officers or employees acting as part of their official duties; on a worldwide, perpetual, irrevocable, royalty-free basis to BMJ Publishing Group Ltd ("BMJ") its licensees and where the relevant Journal is co-owned by BMJ to the co-owners of the Journal, to publish the Work in this journal and any other BMJ products and to exploit all rights, as set out in our licence.

The Submitting Author accepts and understands that any supply made under these terms is made by BMJ to the Submitting Author unless you are acting as an employee on behalf of your employer or a postgraduate student of an affiliated institution which is paying any applicable article publishing charge ("APC") for Open Access articles. Where the Submitting Author wishes to make the Work available on an Open Access basis (and intends to pay the relevant APC), the terms of reuse of such Open Access shall be governed by a Creative Commons licence – details of these licences and which Creative Commons licence will apply to this Work are set out in our licence referred to above.

Other than as permitted in any relevant BMJ Author's Self Archiving Policies, I confirm this Work has not been accepted for publication elsewhere, is not being considered for publication elsewhere and does not duplicate material already published. I confirm all authors consent to publication of this Work and authorise the granting of this licence.

For peer review only

Manuscript Title:

Longitudinal symptom profile of palliative care patients receiving a nurse-led end of life (PEACH) Program to support preference to die at home

Corresponding author (Full name, postal address and e-mail):

Professor Josephine Sau Fan Chow

Address: 1 Campbell Street, Liverpool NSW 1871

E mail: Josephine.Chow@health.nsw.gov.au

Full name, department, institution, city and country of all co-authors:

Meera R Agar,[1,2] Wei Xuan,[3] Jessica Lee,[4,5] Greg Barclay,[6] Alan Oloffs,[7] Kim Jobburn,[8] Janeane Harlum,[2] Nutan Maurya,[9] Josephine Sau Fan Chow[9,10,11]

[1]Centre for Improving Palliative, Aged and Chronic Care through Clinical Research and Translation (IMPACCT) & Faculty of Health, University of Technology Sydney, Sydney, New South Wales, Australia

[2]Department of Palliative Care, Cancer Services South Western Sydney Local Health District, Liverpool, Sydney, New South Wales, Australia.

[3]South Western Sydney Clinical School, University of New South Wales, Liverpool, Sydney, New South Wales, Australia.

[4]Concord Centre for Palliative Care, Concord Repatriation General Hospital, Sydney, New South Wales, Australia

[5]Concord Clinical School, University of Sydney, New South Wales, Australia.

For peer review only - http://bmjopen.bmj.com/site/about/guidelines.xhtml

[6]Palliative care services, Illawarra Shoalhaven Local Health District, New South Wales, Australia.

[7]Nepean Cancer Centre, Nepean Hospital, Nepean Blue Mountains Local Health District, Penrith, New South Wales, Australia.

[8]Clinical Innovation and Business Unit, South Western Sydney Local Health District, Liverpool, Sydney, New South Wales, Australia.

[9]South Western Sydney Nursing & Midwifery Research Alliance, Ingham Institute Applied Medical Research, Liverpool, Sydney, New South Wales, Australia

[10]Faculty of Medicine, University of New South Wales, Sydney, New South Wales, Australia

[11]NICM Health Research Institute, Western Sydney University, New South Wales, Australia.

For peer review only

For peer review only - http://bmjopen.bmj.com/site/about/guidelines.xhtml

ABSTRACT

Objectives: Tailored models of home-based palliative care aimed to support death at home, should also ensure optimal symptom control. This study aimed to explore symptom occurrence and distress over time in Palliative Extended And Care at Home (PEACH) model of care recipients.

Design: This was a prospective cohort study

Setting and Participants: Participants were consecutive recipients of the PEACH rapid response nurse-led model of care in metropolitan Sydney (Dec 2013-Jan 2017) who were in the last weeks of life with a terminal or deteriorating phase of illness and had a preference to be cared or die at home.

Outcome Measures: De-identified data including sociodemographic and clinical characteristics, and symptom distress scores (Symptom Assessment Score) were collected at each clinical visit. Descriptive statistics and forward selection logistic regression analysis were used to explore influence of symptom distress levels on mode of separation [(1) died at home while still receiving a PEACH package, (2) admitted to a hospital or an inpatient palliative care unit or (3) discharged from the package (alive and no longer requiring PEACH)] across 4 symptom distress level categories.

Results: 1,754 consecutive clients received a PEACH package (Mean age 70 years, 55% male). 75.7% (n=1327) had a home death, 13.5% (n=237) were admitted and 10.8% (n=190) were still alive and residing at home when the package ceased. Mean symptom distress scores improved from baseline to final scores in the three groups (P<0.0001). The frequency of no symptom distress score (0) category was higher in the home death group. Higher scores for

For peer review only - http://bmjopen.bmj.com/site/about/guidelines.xhtml

nausea, fatigue, insomnia, and bowel problems were independent predictors of who was admitted.

Conclusion: Tailored home-based palliative care models to meet preference to die at home, achieve this whilst maintaining symptom control. A focus on particular symptoms may further optimise these models of care.

Strengths & limitations of this study

- This study is one of the largest cohorts of a tailored model of care aimed at improving the rates of home death for those where it was their expressed preference.
- The symptom assessments were collected as part of routine clinical care using a reliable and valid measure designed to be integrated into routine clinical care.
- Only symptoms assessed by the Symptom Assessment Scale were included in the analysis.

For peer review only

For peer review only - http://bmjopen.bmj.com/site/about/guidelines.xhtml

BACKGROUND

Symptom burden is one of the most distressing aspects of the end of life (EOL) experience for people with a palliative diagnosis and for their families and loved ones[1, 2]. The symptoms which are prevalent near the EOL are well documented and include breathlessness and respiratory secretions, pain, fatigue, anorexia, and gastrointestinal symptoms[2, 3].

Home-based Palliative care services have been expanding worldwide, and many people at the EOL prefer to be cared for and in many cases also die at home[4-6]. Community palliative care services have been demonstrated to improve patients and caregivers' experience at EOL through better symptom control, coordination of care and improved communication between professionals, patient and family and increase the odds of home deaths[7, 8]. A recent review also suggests the strongest evidence for cost effectiveness relates to home-based interventions through both decreased healthcare costs and resource use and improvements in patient and caregiver outcomes[9] However they also indicated that there is still room to improve home palliative care services and increase benefits to patients, inclusive of those who wish to die at home[8-10]. A study of 25,679 patients who died under the care of a hospital or home-based palliative care team in Australia found that though over 85% of all patients had no severe symptoms prior to death, patients at home had less improvement over all and some symptoms got worse[11].

The PEACH (*Palliative Extended And Care at Home*) model of care (a rapid response nursing care package delivered in conjunction with existing palliative care community services was developed for those who had high or complex palliative care needs to support care at home. Evaluation demonstrated it achieved increased days at home compared to usual care, with the PEACH model of care costs offset by reduced costs due to lower inpatient care needs[12,

For peer review only - http://bmjopen.bmj.com/site/about/guidelines.xhtml

13]. The PEACH model of care was then further tailored to provide EOL care in the last week of life for those patients who had an expressed wish to die at home. A prospective cohort study found that the majority of PEACH recipients with a clear preference to die at home when the service was initiated were able to die at home[14]. Though preference for place of death was achieved it is also important to understand if symptom control was optimal for those who received this model of care.

This study aimed to evaluate symptom occurrence and level of symptom distress over time in recipients of the PEACH model of care, and explore the associations of symptom distress with mode of separation.

METHODS

Study Design

Prospective cohort study. Ethical approval was granted from South Western Sydney Local Health District Human Research Ethics Committee (LNR/15/LPOOL/591) and Silver Chain Human Research Ethics Committee (EC App 144).

Study Population

Patients registered with the specialist palliative care services in five Local Health Districts (LHDs) in metropolitan Sydney, were eligible to receive a PEACH package if they were assessed as likely to be in the last week of life (terminal or deteriorating phase of illness[15] and Australia-modified Karnofsky Performance Scale (AKPS) score ≤40[16], have burdensome symptoms and/or require increased level of support, and had a preference to die at home. Consecutive recipients of the PEACH package between December 2013 and January 2017 were included.

For peer review only - http://bmjopen.bmj.com/site/about/guidelines.xhtml

For peer review only

Patient and public involvement

Patients or the public were not involved in the design, or conduct, or reporting, or dissemination plans of our research.

Study Setting

The PEACH Program is coordinated through one LHD in metropolitan Sydney, Australia to provide packages across five New South Wales metropolitan LHDs (South Western Sydney, Illawarra Shoalhaven, Nepean Blue Mountains, Sydney and Western Sydney) with PEACH nursing services delivered in partnership with a non-government organisation (Silver Chain Group). Australia provides universal healthcare (Medicare), which may be supplemented by private health insurance.

Study intervention

The PEACH Program commenced in 2013 funded by the New South Wales Government, Australia in the participating health districts to address the needs of the community with a palliative diagnosis to facilitate a chosen home death while maintaining quality in the EOL care[14]. PEACH enables clients in their deteriorating and/or terminal phase to die at home or to stay at home as long as possible as per their wishes through provision of intensive, rapid, flexible, individualised nurse -led care and coordination for EOL care (last 7 days or can be extended) while maintaining satisfactory symptoms control and function at home.

Patients who were undecided about their preferred place of death but expressed an interest in attempting to stay home for EOL care or who wished to remain at home and transfer to hospital when death was imminent were also provided with a PEACH package. It was recognised for some clients there are a number of factors such as confidence in ability to die

For peer review only - http://bmjopen.bmj.com/site/about/guidelines.xhtml

at home by either the patient and/or carer as well as cultural, religious, education or family demographics such as younger children in the home, impacted on preference for EOL care, i.e. undecided or transfer when death was imminent. Achieving end of life care preference was considered as one of the main objectives of the Program.

The PEACH package is centrally coordinated through a hub which coordinates community care services including community palliative care. The package provides some services in addition to existing services delivered by General Practitioners, Community Nurses and Specialist Palliative Care Teams. The additional services were provided by a PEACH assistant in nursing during day time to provide either personal care or some respite to the carer for up to 1 hour per day, 7 days per week, and a PEACH registered nurse visit in the evening and overnight telephone or video support by an experienced palliative care nurse who were fully briefed of the patient's care plan until the client separated from the Program.

Data collection and variables

Data was collected routinely for all consecutive PEACH package recipients until separation from the package. De-identified data were used from administrative and clinical information systems obtaining the prospectively collected standardized data requirements and clinical assessments which were a requirement at referral to the PEACH program and during delivery of the PEACH package. To ensure confidentiality and anonymity data was collected using an alpha-numeric code for each participant.

Symptom occurrence and symptom distress

For peer review only - http://bmjopen.bmj.com/site/about/guidelines.xhtml

All patients as per the model of care were in receipt of daily clinical visits, which included a daily symptom score assessment until separation from the program. At times some clients/carers declined the daily visit and hence SAS would not be recorded for that day.

Symptom distress was assessed using Symptom Assessment Scale (SAS), which measures the perceived distress from common symptoms experienced by people receiving palliative care [17]. It is a valid and reliable patient reported outcome measure suitable for routine clinical care with patients requiring palliative and EOL[17, 18] in Australia. The scale assessed eight symptom dimensions: pain, insomnia (difficulty sleeping), nausea, vomiting, bowel problems, appetite problems, breathing problems, and fatigue on a 11 point numerical scale with zero being no distress due to that symptom and 10 being the worst distress possible. Symptom distress was categorized into four levels based on the score for each symptom dimension (0, >0 - ≤3, >3 - ≤6 and >6 - ≤10).

Mode of separation

Mode of separation, which was defined as the status of the patient at the end of the PEACH package episode of care (discharge, transfer or death) and the place to which the person is transferred[19]. Three possible modes of separation were identified from the PEACH package, namely i) person died at home whilst still receiving a PEACH package, ii) was admitted to a hospital or inpatient palliative care unit or if iii) were discharged from the package (alive and no longer requiring PEACH).

Socio-demographic and clinical variables

At the time of referral to the PEACH program socio-demographic clinical data was collected including age, gender, caregiver relationship to client (spouse, child, grandchild, other

For peer review only - http://bmjopen.bmj.com/site/about/guidelines.xhtml

relative, others), preference for place of death on referral (classified as home, hospital/Palliative Care Unit, or undecided), diagnosis (malignant, non-malignant), Palliative Care Phase on referral, location of referral (hospital/Palliative Care Unit, community), language spoken (English, Some English, no English), and as a proxy measure of geographical distance to admitted hospital services Local Government Area (metropolitan, inner and outer regional, remote or very remote as defined by Australian Classification of Local Governments (ACLG) determined by the Australian Bureau of Statistics (ABS)).

Statistical analyses

Data was analysed using SAS software (Version 9.4). Clinical and demographic characteristics were summarised using frequency counts and percentages for categorical data and the mean and standard deviation (SD) for continuous variables.

Mean Symptom Assessment Scale Scores at baseline (on referral) and final (average of the two scores recorded prior to separation) were calculated and compared for each symptom by the frequency and four levels of symptom distress by mode of separation (PEACH recipients who died at home versus those who were admitted to hospital or Palliative Care Unit as mode of separation). Forward selection logistic regression analysis were used to explore influence of symptom distress levels on mode of separation across the four symptom distress level categories.

For peer review only - http://bmjopen.bmj.com/site/about/guidelines.xhtml
For peer review only

RESULTS

Recipients of the PEACH model of care

A total of 1,754 consecutive palliative care patients (Table 1) received a PEACH package across the 5 LHDs from December 2013 to January 2017.

The full participant characteristics are described elsewhere[14] but in summary the majority had a cancer diagnosis, mean age was 70 years (0-106), and 55.4 % (n=978) of them were male. Most patients reported their preferred place of death to be home (89%, n=1561) with smaller numbers citing the hospital or Palliative Care Unit (3.8%, n=67), and 7.2% (n=126) of the clients were not sure about their preferences. Overall, 75.7% (n=1327) clients died at home, 13.5% (n=237) were admitted to hospital or PCU and 10.8% (N=190) were alive at the end of the episode of care from PEACH program.

Almost, 78.7% of the clients who indicated they preferred to die at home met their wish, 55.6 % of clients who were unsure/undecided of preferred place of death on referral to the Program died at home (Table 1).

The duration of stay on the program for clients ranged from <24 hrs to 2 months. The median duration on the program was 6 days. The duration of the stay was same irrespective of the mode of separation.

Patients who were discharged and came back onto the program at a later date, they were counted as another patient encounter. Thirty-three clients who were discharged from the Program re-joined later.

For peer review only - http://bmjopen.bmj.com/site/about/guidelines.xhtml

Table 1: Socio-demographic characteristics at referral to the PEACH program by the three modes of separation

	Died at home (N=1327)	Admitted to Hospital OR Palliative Care Unit (N=237)	Alive and no longer requiring PEACH (N=190)
**Age, Mean (SD)**	70.8 (14.7)	69.3 (14.2)	68.4 (18.0)
**Gender (n, %)**			
Female	605 (45.6)	96 (40.5)	83 (43.7)
Male	722 (54.4)	141 (59.5)	107 (56.3)
**Diagnostic category (n, %)**			
Cancer	1094 (82.5)	214 (90.3)	146 (76.8)
Non-cancer	232 (17.50)	23 (9.7)	44 (23.2)
**Referral source (n, %)**			
Community Health Centre	1014 (76.4)	174 (73.4)	130 (68.4)
Hospital/Palliative Care Unit – Ward/Others	313 (23.6)	63 (26.6)	60 (31.6)
**Language (n, %)**			
English	1126 (84.9)	207 (87.3)	160 (84.2)
Some English	77 (5.8)	13 (5.5)	8 (4.2)
Non-English speaking	124 (9.3)	17 (7.2)	22 (11.6)
**Preferred place of death (n, %)**			
Home	1229 (92.6)	181 (76.4)	151 (79.5)
Hospital/Palliative Care Unit	28 (2.1)	25 (10.6)	14 (7.4)
Undecided	70 (5.3)	31 (13.1)	25 (13.2)
**LGA category (n, %)**			
Major city	1013 (76.3)	195 (82.3)	154 (81.1)
Inner regional	288 (21.7)	39 (16.5)	35 (18.4)
Outer regional	26 (2.0)	3 (1.3)	1 (0.5)
**Carer relationship (n, %)**			
Spouse	657 (50.0)	150 (63.8)	97 (51.6)
Child/Grand Child	517 (39.4)	67 (28.5)	70 (37.2)
Others	96 (7.3)	11 (4.7)	16 (8.5)
Sibling	43 (3.3)	7 (3.0)	5 (2.7)

Symptom distress over time by mode of separation

In the group who died at home, the mean symptom distress scores all improved remaining in the mild symptom distress category, though fatigue showed the least improvement and was rated as moderate levels of symptom distress (Table 2). A similar pattern was seen for those who were admitted to a hospital or palliative care unit, or who were discharged from the PEACH package except for fatigue which worsened in both groups.

For peer review only - http://bmjopen.bmj.com/site/about/guidelines.xhtml

When the symptom distress scores were compared for the three modes of separation, no significant differences in the baseline score was observed by mode of separation . However a significant differences in the symptom distress scores prior to separation (final score) was observed for all symptoms by mode of separation (Table 2).

Table 2: Comparison of baseline and final symptom distress scores by mode of separation

	Died at home N=1327		Admitted to Hospital of Palliative Care Unit N=237		Alive and no longer requiring PEACH N=190		
**Baseline score**	**Mean**	**SD**	**Mean**	**SD**	**Mean**	**SD**	**p-value**
Insomnia	1.47	2.12	1.64	2.26	1.33	1.84	0.322
Appetite	2.16	2.74	2.37	2.79	2.45	2.78	0.297
Nausea	1.41	2.50	1.45	2.35	1.47	2.39	0.933
Bowel Problems	1.55	2.28	1.49	2.32	1.39	2.11	0.651
Breathing Problems	2.06	2.56	1.96	2.53	2.19	2.64	0.666
Fatigue	5.12	3.26	4.76	3.08	4.92	3.09	0.271
Pain score	1.89	2.26	1.84	2.36	1.60	2.07	0.477
Vomiting score	0.30	1.11	0.44	1.36	0.43	1.11	0.311
**Final score**	**Mean**	**SD**	**Mean**	**SD**	**Mean**	**SD**	**p-value**
Insomnia	0.69	1.69	1.24	2.21	0.73	1.58	<.0001
Appetite	1.05	2.58	1.75	2.55	1.25	2.09	0.0006
Nausea	0.18	0.87	0.51	1.49	0.52	1.46	<.0001
Bowel Problems	0.79	1.73	1.35	2.13	0.76	1.58	<.0001
Breathing Problems	2.00	2.41	1.35	2.29	1.09	1.76	<.0001
Fatigue	4.98	4.03	6.06	2.49	5.36	2.66	0.0002
Pain score	1.59	2.11	1.91	2.54	1.15	1.99	0.0018
Vomiting score	0.12	0.73	0.30	1.34	0.19	0.86	0.0112

The final symptom distress scores by the four categories (score =0, >0 - ≤3, >3 - ≤6 and >6 - ≤10) for each symptom for the two modes of separation (died at home, admitted) is outlined

For peer review only - http://bmjopen.bmj.com/site/about/guidelines.xhtml

in Table 3. The other 3 categories (>0 - ≤3, >3 - ≤6 and >6 - ≤10) were compared to the =0 category. The frequency of no symptom distress score (0) category was higher in the group who died at home, as compared to the group who were admitted to hospital. The higher score category for nausea, fatigue, insomnia and bowel problems were associated with being admitted to hospital.

For peer review only

Table 3: Final symptom distress by category for those who died at home and those who were admitted

	Symptom distress category (score range)	Frequency, N (%)		P value
		Died at home	Admitted	
**Insomnia**		N=1321	N=237	
	=0	942 (87.1)	139 (12.9)	
	>0 - ≤3	266 (79.2)	70 (20.8)	0.0006
	>3 - ≤6	96 (82.1)	21 (17.9)	
	>6 - ≤10	17 (70.8)	7 (29.2)	
**Appetite**		N=1271	N=235	
	=0	880 (89.6)	102 (10.4)	
	>0 - ≤3	224 (71.8)	88 (28.2)	<.0001
	>3 - ≤6	106 (76.3)	33 (23.7)	
	>6 - ≤10	61 (83.6)	12 (16.4)	
**Nausea**		N=1318	N=237	
	=0	1182 (86.8)	180 (13.2)	
	>0 - ≤3	112 (72.3)	43 (27.7)	<.0001
	>3 - ≤6	19 (65.5)	10 (34.5)	
	>6 - ≤10	5 (55.6)	4 (44.4)	
**Bowel problem**		N=1322	N=237	
	=0	836 (88.3)	111 (11.7)	
	>0 - ≤3	370 (79.4)	96 (20.6)	<.0001
	>3 - ≤6	97 (80.8)	23 (19.2)	
	>6 - ≤10	19 (73.1)	7 (26.9)	

For peer review only - http://bmjopen.bmj.com/site/about/guidelines.xhtml

**Breathing Problem**		N=1323	N=237	
	=0	504 (79.0)	134 (21.0)	
	>0 - ≤3	542 (90.2)	59 (9.8)	<.0001
	>3 - ≤6	219 (85.6)	37 (14.5)	
	>6 - ≤10	58 (89.2)	7 (10.8)	
**Fatigue**		N=1311	N=237	
	=0	261 (98.5)	4 (1.5)	
	>0 - ≤3	86 (76.1)	27 (23.9)	<.0001
	>3 - ≤6	314 (80.9)	74 (19.1)	
	>6 - ≤10	650 (83.1)	132 (16.9)	
**Pain**		N=1324	N=237	
	=0	469 (84.7)	85 (15.3)	
	>0 - ≤3	635 (85.7)	106 (14.3)	0.0290
	>3 - ≤6	195 (85.2)	34 (14.9)	
	>6 - ≤10	25 (67.6)	12 (32.4)	
**Vomiting**		N=1320	N=237	
	=0	1246 (85.5)	212 (14.5)	
	>0 - ≤3	54 (74.0)	19 (26.0)	0.0103
	>3 - ≤6	16 (84.2)	3 (15.8)	
	>6 - ≤10	4 (57.1)	3 (42.9)	

Note – frequency presented row wise.

Association between symptom distress and mode of separation

On univariate analyses the higher the final symptom distress score (average of last two scores recorded prior to separation), the higher the odds of patient being admitted to hospital or palliative care unit as mode of separation, except for breathing problems which showed the reverse association (Table 4).

Table 4. Univariate analyses of symptom distress score by symptom for mode of separation (admitted to hospital or palliative care unit versus died at home)

Symptom score	OR	95%CI	p-value
Insomnia	1.13	1.048 - 1.221	0.0015
Appetite	1.11	1.048 - 1.168	0.0003
Nausea	1.31	1.178 - 1.465	<.0001
Bowel	1.13	1.054 - 1.218	0.0007
Breathing Problem	0.88	0.812 - 0.945	0.0006
Fatigue	1.09	1.041 - 1.144	0.0003
Pain	1.05	0.973 - 1.126	0.2175
Vomiting	1.21	1.063 - 1.377	0.0040

On multivariate analysis with forward selection higher final symptom distress scores for nausea, fatigue, insomnia and bowel problems were associated with being admitted to hospital (Table 5).

Table 5: Multivariate analyses (with forward selection) of symptom distress score by symptom for mode of separation (admitted to hospital or palliative care unit versus died at home)

For peer review only

Symptom score	OR	95%CI	*p-value
(1) Nausea	1.23	1.10 - 1.38	0.0003
(2) Breathing	**0.86**	**0.79 -0.93**	0.0001
(3) Fatigue	1.08	1.03 -1.14	0.0029
(4) Insomnia	1.10	1.01 - 1.20	0.0226
(5) Bowel	1.09	1.02 - 1.19	0.0187

*with the adjustment of preferred place of death and diagnostic category

DISCUSSION

This study has demonstrated that a tailored model of care to support palliative care patients who have a preference to die at home, supports excellent symptom control with symptom distress of mild severity and improving over time regardless of mode of separation from the PEACH package, apart from fatigue. The frequency of a rating of no symptom distress was highest in the group who died at home. The higher symptom distress for nausea, fatigue, insomnia and bowel problems were associated with being admitted to hospital. Symptom distress due to breathing problems were less likely to be admitted to hospital possibly reflecting the breathing changes in the imminently dying person where moving location of care would be inappropriate.

These findings are in the context of our prior study[14] finding that the majority of PEACH package recipients who have a clear preference to die at home when the service is initiated, were able to achieve their goal to die at home. People who were undecided at referral or had a preference for admission when death was imminent were more likely to be admitted to hospital or a PCU than die at home. People with a non-cancer diagnosis or who were cared

For peer review only - http://bmjopen.bmj.com/site/about/guidelines.xhtml

for by their child or grandchild or friend/relative as caregiver were less likely to be admitted to hospital/PCU as a mode of separation.

We found that higher symptom distress score increases the likelihood of patient being admitted to the hospital or palliative care unit. This mirrors the findings of other studies which have reported that patients were more likely to be hospitalized or die in the hospital if they had higher functional status or greater pain intensity (pain score ≥ 2)[20] or when symptoms such as pain and dyspnoea were present[21]. A recent home-based palliative care cohort study of adult cancer patients in Singapore showed that high symptom needs increased incidence rate ratios of acute healthcare utilization[22]. The reason that the patients sought admission to the hospital or palliative care unit could be due to concerns in managing symptoms at home which might require a level of supervision and physical assistance beyond caregivers' coping capacity, or to seek clinical assessment and adjustment to management, with these decisions may have been supported by the clinicians involved in their care in some cases.

Studies demonstrate a significant decrease in the level of severe symptom distress after start of palliative care [11, 23]. Notably Gill and colleagues[23] found no change in the rates of fatigue ascertained from monthly interviews of 665 decedents of community living older people, and symptom that we also found caused moderate symptom distress increasing over time. Eagar and colleagues evaluated data derived from the Australian Palliative Care Outcomes Collaborative and found for 25,679 patients who died under the care of a hospital or home based palliative care team symptom distress improved and over 85% of patients had no severe symptom distress (scores 8 -10) just prior to death[11].[1] Similarly they found high

For peer review only - http://bmjopen.bmj.com/site/about/guidelines.xhtml

rates for severe symptom distress for fatigue with this occurring in 14.6% of those in hospital and 10% of those at home just before death.

There is limited literature exploring differences in severe symptom outcomes for palliative care patients receiving hospital care compared with those receiving care at home. Some studies report that symptom outcomes are better for hospital patients and patients at home have less improvement overall and some symptoms get worse. A retrospective cohort study of 359 advanced cancer patients reported that those with final place of care as home had the lowest pain and depression scores as compared to patients in the inpatient hospice group or the hospital group[20]. A study in China found enhanced intensity of home palliative care visits with two additional days per week and formulated standard operating procedures for dyspnoea could significantly reduce the rate of ED visits by 30.7% ($P < 0.05$) due to dyspnoea during the last 6 months of life[24]. Nausea and well-being scores were also significantly worse in the inpatient hospice group, compared to the home group[20]. It is not clear if those who were hospitalised had access to specialist palliative care. In contrast, Eagar and colleagues found that palliative care patients under the palliative care teams in the hospital were 3.7 times less likely than those at home to have no severely distressing symptoms before death[11]. This suggests that supplementing specialist palliative care with bespoke packages for the last week of life can ensure severe distress from symptoms is negated.

Limitations

This was a prospective cohort study and there is no usual care comparator, and the model was delivered in the Australian health care context which may limit generalizability of this model elsewhere. The majority of the recipients had cancer.

For peer review only - http://bmjopen.bmj.com/site/about/guidelines.xhtml

In this study, only symptoms assessed by the Symptom Assessment Scale were included in the analysis, there is possibility that patients might have other prominent or bothersome symptoms which may be unique to a particular palliative diagnosis that could have resulted in separation from the program.

As the data collection ceased at separation from the package, the ongoing or change in symptom distress until death for patients admitted to hospital was unknown, though it is likely that these patients died during that index admission.

Recommendations

When designing home based palliative care models aimed to support palliative care patients at home, the model needs to be tailored to particular needs, time points in the illness trajectory and preferences and evaluated to ensure ongoing capacity to refine the model to optimise patient outcomes. Further studies need to include more participants with non-cancer diagnoses. There is opportunity to further intensify support and symptom management when symptom distress is not improving or increasing, and fatigue warrants specific attention.

Conclusion

This study findings suggest that not only does the PEACH model of care allow people with palliative diagnoses to meet their preference to die at home, it achieves this whilst maintaining symptom control. Response to increase in particular symptoms may further optimise these models of care.

Ethics Statement

For peer review only - http://bmjopen.bmj.com/site/about/guidelines.xhtml

Patient consent for publication

Not required.

Ethics Approval

Ethical approval was granted from South Western Sydney Local Health District Human Research Ethics Committee (LNR/15/LPOOL/591) and Silver Chain Human Research Ethics Committee (EC App 144).

Meera R Agar,[1,2] Wei Xuan,[3] Jessica Lee,[4, 5] Greg Barclay,[6] Alan Oloffs,[7] Kim Jobburn,[8] Janeane Harlum,[2] Nutan Maurya,[8] Josephine Sau Fan Chow[8]

Contributorship Statement

MA, JC, WX, NM developed the idea and methodology of the study, provided governance and oversight and draft manuscript. JC, KJ, JH helped conduct the study, reviewed draft manuscripts. JC and KJ provided support and guidance of staff. MA, WX, NM analysed and interpreted the data. JL, GB, AO provided advice about study planning. JC, MA, WX and NM drafted the protocol and manuscript. All authors approved the final version of this manuscript.

Competing interests

The authors have no financial or professional relationships which may pose a competing interest.

Funding

For peer review only - http://bmjopen.bmj.com/site/about/guidelines.xhtml

This research received no specific grant funding from any funding agency in the public, commercial, or not-for-profit sectors.

Data sharing statement

Data for this study can be provided upon request.

For peer review only

REFERENCES:

1. Baillie J, Anagnostou D, Sivell S, et al. Symptom management, nutrition and hydration at end-of-life: a qualitative exploration of patients', carers' and health professionals' experiences and further research questions. BMC Palliative Care 2018;17(1):60. doi: 10.1186/s12904-018-0314-4
2. Singer AE, Meeker D, Teno JM, et al. Symptom trends in the last year of life from 1998 to 2010: a cohort study. Ann Intern Med 2015;162(3):175-83. doi: 10.7326/M13-1609
3. Kehl KA, Kowalkowski JA. A systematic review of the prevalence of signs of impending death and symptoms in the last 2 weeks of life. Am J Hosp Palliat Care 2013;30(6):601-16. doi: 10.1177/1049909112468222 [published Online First: 20121212]
4. Hoare S, Morris ZS, Kelly MP, et al. Do Patients Want to Die at Home? A Systematic Review of the UK Literature, Focused on Missing Preferences for Place of Death. PLoS One 2015;10(11):e0142723. doi: 10.1371/journal.pone.0142723 [published Online First: 2015/11/12]
5. Nysæter TM, Olsson C, Sandsdalen T, et al. Preferences for home care to enable home death among adult patients with cancer in late palliative phase – a grounded theory study. BMC Palliative Care 2022;21(1):49. doi: 10.1186/s12904-022-00939-y
6. Cai J, Zhang L, Guerriere D, et al. Where Do Cancer Patients in Receipt of Home-Based Palliative Care Prefer to Die and What Are the Determinants of a Preference for a Home Death? Int J Environ Res Public Health 2020;18(1):235. doi: 10.3390/ijerph18010235
7. Higginson IJ, Sarmento VP, Calanzani N, et al. Dying at home--is it better: a narrative appraisal of the state of the science. Palliat Med 2013;27(10):918-24. doi: 10.1177/0269216313487940 [published Online First: 2013/05/24]

For peer review only - http://bmjopen.bmj.com/site/about/guidelines.xhtml

8. Shepperd S, Goncalves-Bradley DC, Straus SE, et al. Hospital at home: home-based end-of-life care. Cochrane Database Syst Rev 2021;3:CD009231. doi: 10.1002/14651858.CD009231.pub3 [published Online First: 20210316]

9. Adamson E, Cruickshank S. A 'good death' at home: community nurses helping to make it possible. Br J Community Nurs 2013;18(1):40-2. doi: 10.12968/bjcn.2013.18.Sup9.S40 [published Online First: 2013/01/10]

10. Gomes B, Calanzani N, Higginson IJ. Benefits and costs of home palliative care compared with usual care for patients with advanced illness and their family caregivers. Jama 2014;311(10):1060-1. doi: 10.1001/jama.2014.553 [published Online First: 2014/03/13]

11. Eagar K, Clapham SP, Allingham SF. Palliative care is effective: but hospital symptom outcomes superior. BMJ Support Palliat Care 2020;10(2):186-90. doi: 10.1136/bmjspcare-2018-001534 [published Online First: 20180831]

12. McCaffrey N, Agar M, Harlum J, et al. Is home-based palliative care cost-effective? An economic evaluation of the Palliative Care Extended Packages at Home (PEACH) pilot. BMJ Support Palliat Care 2013;3(4):431-5. doi: 10.1136/bmjspcare-2012-000361 [published Online First: 2014/06/21]

13. McCaffrey N, Agar M, Harlum J, et al. Better informing decision making with multiple outcomes cost-effectiveness analysis under uncertainty in cost-disutility space. PLoS One 2015;10(3):e0115544. doi: 10.1371/journal.pone.0115544 [published Online First: 2015/03/10]

14. Agar MR, Xuan W, Lee J, et al. Factors Associated With Mode of Separation for People With Palliative Diagnoses With Preference for Home Death Receiving Care From a

For peer review only - http://bmjopen.bmj.com/site/about/guidelines.xhtml

For peer review only

Nurse-Led End of Life (Palliative Extended and Care at Home) Program. Journal of Hospice & Palliative Nursing 9000

15. Eagar K, Gordon R, Green J, et al. An Australian casemix classification for palliative care: lessons and policy implications of a national study. Palliat Med 2004;18(3):227-33. doi: 10.1191/0269216304pm876oa
16. Abernethy AP, Shelby-James T, Fazekas BS, et al. The Australia-modified Karnofsky Performance Status (AKPS) scale: a revised scale for contemporary palliative care clinical practice [ISRCTN81117481]. BMC Palliat Care 2005;4:7. doi: 10.1186/1472-684X-4-7 [published Online First: 20051112]
17. Aoun SM, Monterosso L, Kristjanson LJ, McConigley R. Measuring symptom distress in palliative care: psychometric properties of the Symptom Assessment Scale (SAS). J Palliat Med. 2011 Mar;14(3):315-21. doi: 10.1089/jpm.2010.0412
18. Daveson BA, Allingham SF, Clapham S, et al. The PCOC Symptom Assessment Scale (SAS): A valid measure for daily use at point of care and in palliative care programs. PLoS One 2021;16(3):e0247250. doi: 10.1371/journal.pone.0247250 [published Online First: 20210325]
19. Australian Institute of Health and Welfare (AIHW). Mode of Separation Definition, National health data dictionary version 16. 2012. Accessed June 2022. https://www.aihw.gov.au/getmedia/a90ff140-8abf-4e30-8861-da2ebf368d35/14306.pdf.aspx?inline=true.
20. Tay RY, Choo RWK, Ong WY, et al. Predictors of the final place of care of patients with advanced cancer receiving integrated home-based palliative care: a retrospective cohort study. BMC Palliat Care 2021;20(1):164. doi: 10.1186/s12904-021-00865-5 [published Online First: 20211018]

For peer review only - http://bmjopen.bmj.com/site/about/guidelines.xhtml

21. Okamoto Y, Fukui S, Yoshiuchi K, et al. Do Symptoms among Home Palliative Care Patients with Advanced Cancer Decide the Place of Death? Focusing on the Presence or Absence of Symptoms during Home Care. J Palliat Med 2016;19(5):488-95. doi: 10.1089/jpm.2015.0184 [published Online First: 2016/02/06]

22. Zhuang Q, Chong PH, Ong WS, et al. Longitudinal patterns and predictors of healthcare utilization among cancer patients on home-based palliative care in Singapore: a group-based multi-trajectory analysis. BMC Med. 2022;20(1):313. Published 2022 Sep 22. doi:10.1186/s12916-022-02513-y

23. Gill TM, Gahbauer EA, Leo-Summers L, et al. Trends in Restricting Symptoms at the End of Life from 1998 to 2019: A Cohort Study of Older Persons. J Am Geriatr Soc 2021;69(2):450-58. doi: 10.1111/jgs.16871 [published Online First: 20201103]

24. Hsu H-S, Wu T-H, Lin C-Y, et al. Enhanced home palliative care could reduce emergency department visits due to non-organic dyspnea among cancer patients: a retrospective cohort study. BMC Palliative Care 2021;20(1):42. doi: 10.1186/s12904-021-00713-6

For peer review only

For peer review only - http://bmjopen.bmj.com/site/about/guidelines.xhtml

STROBE Statement—Checklist of items included in the manuscript

	Item No	Recommendation	Page
**Title and abstract**	1	(*a*) Indicate the study's design with a commonly used term in the title or the abstract	3
		b) Provide in the abstract an informative and balanced summary of what was done and what was found	3
**Introduction**			
Background/rationale	2	Explain the scientific background and rationale for the investigation being reported	6
Objectives	3	State specific objectives, including any prespecified hypotheses	7
**Methods**			
Study design	4	Present key elements of study design early in the paper	8
Setting	5	Describe the setting, locations, and relevant dates, including periods of recruitment, exposure, follow-up, and data collection	8-9
Participants	6	(*a*) Give the eligibility criteria, and the sources and methods of selection of participants. Describe methods of follow-up<br>(*b*) For matched studies, give matching criteria and number of exposed and unexposed	8
Variables	7	Clearly define all outcomes, exposures, predictors, potential confounders, and effect modifiers; give diagnostic criteria, if applicable	10
Data sources/measurement	8*	For each variable of interest, give sources of data and details of methods of assessment (measurement); describe comparability of assessment methods if there is more than one group	10-11
Bias	9	Describe any efforts to address potential sources of bias	-
Study size	10	Explain how the study size was arrived at	10
Quantitative variables	11	Explain how quantitative variables were handled in the analyses; if applicable, describe which groupings were chosen and why	10-11
Statistical methods	12	a) Describe all statistical methods, including those used to control for confounding<br>b) Describe any methods used to examine subgroups and interactions<br>c) Explain how missing data were addressed<br>d) Cohort study - if applicable, explain how loss to follow-up was addressed<br>e) Describe any sensitivity analyses	12
**Results**			
Participants	13*	a) Report numbers of individuals at each stage of study - e.g., numbers potentially eligible, examined for eligibility, confirmed eligible,	13

For peer review only

		included in the study, completing follow-up, and analyzed<br>b) Give reasons for nonparticipation at each stage.<br>c) Consider use of a flow diagram	
Descriptive data	14*	a) Give characteristics of study participants (e.g., demographic, clinical, social) and information on exposures and potential confounders<br>b) Indicate number of participants with missing data for each variable of interest<br>c) Summarize follow-up time (e.g., average and total amount)	13
Outcome data	15*	Report numbers of outcome events or summary measures over time	14-17
Main results	16	a) Give unadjusted estimates and, if applicable, confounder-adjusted estimates and their precision (e.g., 95% confidence interval); make clear which confounders were adjusted for and why they were included<br>b) Report category boundaries when continuous variables were categorized<br>c) If relevant, consider translating estimates of relative risk into absolute risk for a meaningful time period	18-23
Other analyses	17	Report other analyses done - e.g., analyses of subgroups and interactions, and sensitivity analyses	-
**Discussion**			
Key results	18	Summarize key results with reference to study objectives	24-26
Limitations	19	Discuss limitations of the study, taking into account sources of potential bias or imprecision; discuss both direction and magnitude of any potential bias	26
Interpretation	20	Give a cautious overall interpretation of results considering objectives, limitations, multiplicity of analyses, results from similar studies, and other relevant evidence	27
Generalizability	21	Discuss the generalizability (external validity) of the study results	26
**Other information**			
Funding	22	Give the source of funding and the role of the funders for the present study and, if applicable, for the original study on which the present article is based	29

For peer review only
